# An integrated method for the identification of novel genes related to oral cancer

**DOI:** 10.1371/journal.pone.0175185

**Published:** 2017-04-06

**Authors:** Lei Chen, Jing Yang, Zhihao Xing, Fei Yuan, Yang Shu, YunHua Zhang, XiangYin Kong, Tao Huang, HaiPeng Li, Yu-Dong Cai

**Affiliations:** 1 School of Life Sciences, Shanghai University, Shanghai, People’s Republic of China; 2 College of Information Engineering, Shanghai Maritime University, Shanghai, People’s Republic of China; 3 Institute of Health Sciences, Shanghai Institutes for Biological Sciences, Chinese Academy of Sciences, Shanghai, People’s Republic of China; 4 Department of Science & Technology, Binzhou Medical University Hospital, Binzhou, Shandong, People’s Republic of China; 5 School of Resources and Environment, Anhui Agricultural University, Hefei, Anhui, People’s Republic of China; 6 CAS Key Laboratory of Computational Biology, CAS-MPG Partner Institute for Computational Biology, Shanghai Institutes for Biological Sciences, Chinese Academy of Sciences, Shanghai, People’s Republic of China; Tianjin University, CHINA

## Abstract

Cancer is a significant public health problem worldwide. Complete identification of genes related to one type of cancer facilitates earlier diagnosis and effective treatments. In this study, two widely used algorithms, the random walk with restart algorithm and the shortest path algorithm, were adopted to construct two parameterized computational methods, namely, an RWR-based method and an SP-based method; based on these methods, an integrated method was constructed for identifying novel disease genes. To validate the utility of the integrated method, data for oral cancer were used, on which the RWR-based and SP-based methods were trained, thereby building two optimal methods. The integrated method combining these optimal methods was further adopted to identify the novel genes of oral cancer. As a result, 85 novel genes were inferred, among which eleven genes (*e*.*g*., MYD88, FGFR2, NF-κBIA) were identified by both the RWR-based and SP-based methods, 70 genes (*e*.*g*., BMP4, IFNG, KITLG) were discovered only by the RWR-based method and four genes (L1R1, MCM6, NOG and CXCR3) were predicted only by the SP-based method. Extensive analyses indicate that several novel genes have strong associations with cancers, indicating the effectiveness of the integrated method for identifying disease genes.

## Introduction

Cancer is a significant public health problem worldwide. Oral cancer (OC) is a subgroup of head and neck cancer; it develops on the lips, tongue, salivary glands, gingiva, oropharynx, and on buccal surfaces [1]. Oral squamous cell carcinoma (OSCC) accounts for more than 90% of all OC [2]. OC is estimated by the WHO (World Health Organization) to be the eleventh most common cancer in the world, accounting for 300,000 new cases and 145,000 deaths in 2012 [3, 4]. The incidence of OC exhibits significant local variation and continues to be high in India, East Asia, Eastern Europe, and parts of South America [5]. Tobacco and alcohol are the most important risk factors for OC [6]. Poor nutrition, genetic factors and viral infection may be potential risk factors for OC [7, 8].

Carcinogenesis is a multi-step process, and a variety of alterations accumulate, driving and gradually increasing tumorigenesis [9]. During the past decade, a large variety of genomic variations has been implicated in OC. EGFR (epidermal growth factor receptor) amplification and over-expression were found in a large proportion of oral tumors [10–12]. ErbB2 amplification and overexpression appear to occur frequently in OC specimens, and high levels of ErbB2 may be related to a worse prognosis of patients [12]. A strong correlation has been detected between c-erbB-2 overexpression and overall survival of patients with oral squamous cell carcinoma [13, 14]. The cyclin family plays a critical role in cell cycle progression. Aberrant up-expression of cyclin D accounts for 36–66% of OC. Amplification and an SNP of cyclin D may be associated with a worse prognosis and susceptibility to OC, respectively [15, 16]. Several lines of evidence suggest that the p53 tumor suppression network is altered in OC [17, 18]. In addition, many other target genes have been reported, such as ras, VEGF (vascular endothelial growth factor), and the MMP (matrix metalloproteinases) family [19–21]. To the best of our knowledge, the mechanism that underlies OC is still unclear. A search for new genes related to OC may facilitate earlier diagnosis and effective treatment.

In general, many standard methods have been used for detection of virulence genes. In the hospital and laboratory, cancer samples are sliced into pathological sections and stained to determine the disease pathology type. Quantitative reverse transcription-PCR is typically used to detect the mRNA level of genes in cancer. IHC (immunohistochemical) and western blotting measure the expression of related proteins in tissues and cells, respectively. However, it is difficult to analyze genes synthetically and comprehensively. In addition, some large-scale experiments have been exploited to screen virulence genes, such as microarray, GWAS (genome-wide association study) and NGS (next-generation sequencing). The most common techniques are time-consuming and costly, and thus new methods must be explored to identify tumor genes. In recent years, with the development of computer techniques [22–28], some of them can be applied to tackle this problem. Up to now, several computational methods have been proposed to identify disease genes. Many of them are based on guilt-by-association [29], i.e., the assumption that genes are similar to their neighbors in a gene network. Thus, the neighbors of the disease genes are more likely to be disease genes. However, these types of methods are local methods that use only part of the network. Thus, they do not always yield good performance. Many other methods employ the Random Walk with Restart (RWR) algorithm to identify disease genes [30–32]. This algorithm simulates a walker starting from a seed node or a set of seed nodes that represent disease genes and random walking on the network. The probability of a node being a disease gene is updated until the probabilities of all nodes become stable. The genes corresponding to nodes with high probabilities are selected as novel candidate disease genes. Recently, computational methods have adopted the shortest path (SP) algorithm to address the problem [33–39]. This algorithm assumes that genes lying in the shortest paths connecting any two disease genes may also be disease genes. Clearly, methods based on RWR or SP algorithms take full advantage of the network compared with those based on guilt-by-association. Thus, they can yield clues for the discovery of novel disease genes.

In this study, we used the RWR and SP algorithms to construct two novel computational methods, the RWR-based method and the SP-based method, respectively, for the identification of novel disease genes. Additionally, an integrated method was constructed by combining these two methods. To indicate the effectiveness of the integrated method, disease genes of OC were employed. It has been reported in some studies that using only the RWR algorithm and SP algorithm consistently produces several false discoveries [33, 40], which may be caused by the structure of the network or lack of consideration of the essential properties of genes. Thus, further rules, produced by the permutation test, associations between candidates and the validation of genes according to their properties, were integrated into the RWR-based and SP-based methods. These methods were executed using the corresponding large network that was built using protein-protein interaction (PPI) information and that was trained on validated OC genes to determine the optimal parameters. The obtained optimal methods were used to infer novel genes related to OC, and the integrated method combined the predicted genes using these two optimal methods to yield 85 novel genes. Among them, eleven genes were obtained by both methods, 70 genes were obtained only by the RWR-based method and four genes were obtained only by the SP-based method. According to the analyses, several genes show stimulative or suppressive effects on cancers by experiments or have a certain relationship with cancers reported in published papers, indicating the utility of the integrated method. It is also clear that the integrated method can provide more comprehensive analysis of various diseases because it is capable of producing more possible disease genes than the RWR-based method or the SP-based method.

## Materials and methods

### Genes related to oral cancer

The OC-related genes were collected from the following three sources: (1) 44 genes were retrieved from UniProt (http://www.uniprot.org/, accessed in July, 2015) [41] after ‘human oral cancer reviewed’ was input as a keyword; (2) seven genes were chosen from the catalogue of oral cancer from the TSGene (https://bioinfo.uth.edu/TSGene/, accessed in July, 2015) database; and (3) 156 genes were retrieved from the NCI (National Cancer Institute, https://gforge.nci.nih.gov, accessed in July, 2015) database using ‘Homo sapiens’ as a keyword. After combining the OC-related genes mentioned above, 202 OC-related genes were finally obtained; these genes are provided in S1 Table. Because our methods are based on the network constructed from the PPI information retrieved from Search Tool for the Retrieval of Interacting Genes/Proteins (STRING) [42], these 202 OC-related genes were mapped into their Ensembl IDs, and those not occurring in the network were discarded. One hundred eighty-four Ensembl IDs of OC-related genes were accessed and composed the set *S*_oc_.

### Protein-protein interactions

PPIs play important roles in several intracellular and intercellular biochemical processes. The known PPI information has been widely used to investigate several protein-related problems, such as protein function prediction [43, 44] and disease gene identification [33, 34, 36, 39]. Based on the results reported in these previous studies, it can be concluded that two proteins that can interact with each other always have a functional relationship. In this study, we attempted to discover novel candidate OC-related genes using validated OC-related genes. Thus, we can utilize PPIs to search for proteins that have a functional relationship with proteins encoded by validated OC-related genes, thereby mining novel OC-related genes.

The PPI information was retrieved from STRING (http://string-db.org/, version 9.1) [42], a well-known online public database that contains known and predicted protein interactions. The interactions reported in STRING are derived from the following four types of sources: (I) Genomic Context; (II) High-throughput Experiments; (III) (Conserved) Coexpression; (IV) Previous Knowledge, which include direct (physical) and indirect (functional) associations between proteins and thus offer more chances to mine hidden protein information. From the database, a file called ‘protein.links.v9.1.txt.gz‘ was retrieved, which contained 2,425,314 human PPIs. Each PPI was represented by two Ensembl IDs and a score ranging between 150 and 999, which indicates the strength of the interaction; i.e., proteins in interactions with higher scores are regarded as more likely to interact with each other. Let us denote the score of a PPI between proteins *p*_1_ and *p*_2_ by *S*(*p*_1_, *p*_2_).

### RWR-based method

RWR is a ranking algorithm [30]. In a network, this algorithm simulates a walker starting from a seed node or a set of seed nodes and random walking on the network. In each step, the probability of the walker walking to each node is updated, and it stabilizes after several steps. Nodes in the network are ranked by the final probabilities assigned to them. Based on the validated disease genes (used as seed nodes), the RWR algorithm can be used to discover novel disease genes by investigating novel genes with high ranks. This algorithm has therefore been used to mine novel disease genes in recent years [30–32]. However, the RWR algorithm consistently provides false discoveries [40], supporting the need for additional screening rules. In this study, we used the RWR algorithm as the basic algorithm and added some rules to build the present RWR-based learning method for the identification of novel OC-related genes. Furthermore, to obtain a better RWR-based method, some parameters were employed that are determined by training the method.

#### Network construction for RWR algorithm

An accurate network is important for the identification of novel OC-related genes. Here, we adopted the PPI information mentioned in the Section “Protein-protein interactions” to build the network. The constructed network defined the 20,770 proteins occurring in the 2,425,314 human PPIs as nodes, and two nodes were adjacent if and only if the corresponding proteins could interact with each other. It can be observed that each edge represented a PPI. To employ the interaction score in the network, each edge was assigned a weight that was defined as the score of the corresponding PPI. For convenience, let us denote the constructed network by *N*_RWR_.

#### Searching for new candidate genes using the RWR algorithm

As mentioned in the Section “Genes related to oral cancer”, a set *S*_oc,_ consisting of 184 Ensembl IDs of OC-related genes was used in this study. The RWR algorithm simulated a random walker starting from *S*_oc_. Before executing the RWR algorithm on *N*_RWR_, each node in *N*_RWR_ was assigned a probability, with 1/184 set for the Ensembl IDs in *S*_oc_ and 0 set for the remaining nodes. The initial probability of each node constituted the probability vector *P*_0_. The RWR algorithm updated the probability vector in each step. Let *P*_*t*_ denote the probability vector after performing the *t*-th step, which can be updated by

$$P_{t+1}=(1-r)A^{T}P_{t}+rP_{0}$$ (1)

where *r* is set to 0.8 and *A* is the column-wise normalized adjacency matrix of *N*_RWR_. The update procedure was repeated until the change between P_*t*_ and *P*_*t*+1_, measured by *L*_1_ norm, was less than 1e-6. Ensembl IDs with high probabilities were considered to be encoded by OC-related genes. Because we did not know which probability was a suitable threshold for selecting candidate genes, we established a parameter, $p_{RWR}^{p}$, for the threshold, which can be determined by training the RWR-based method.

### SP-based method

The SP algorithm is a classic graph algorithm. In recent years, some investigators have applied the SP algorithm for the identification of disease genes [33–39]. The new candidate genes were extracted from the shortest paths connecting any two validated disease genes. Here, we built an SP-based machine learning method to identify novel OC-related genes. Like the RWR-based method, further screening rules were established to discard non-essential candidate genes and select important ones.

#### Network construction for SP algorithm

Similar to *N*_RWR_ for the RWR-based method, we also constructed the network *N*_SP_. As mentioned in the Section “Network construction for RWR-based method”, each edge in *N*_RWR_ represented a PPI. In addition, each PPI had an interaction score as described in the Section “Protein-protein interactions”. This score was used to define the weight of the edge. The range of the interaction score was between 150 and 999, and an edge with a low weight indicated strong correlations between its endpoints in the SP-based model. Thus, for an edge *e* with endpoints *n*_1_ and *n*_2_, weight was defined in the following manner:

$$w(e)=1000-S(p_{1},p_{2})$$ (2)

where *p*_1_ and *p*_2_ were two corresponding proteins of nodes *n*_1_ and *n*_2_. The constructed network *N*_SP_ consisted of 20,770 nodes and 2,425,314 edges.

#### Searching for new candidate genes using the SP algorithm

The SP algorithm, Dijkstra’s algorithm [45], was applied to the network *N*_SP_ to search for all of the shortest paths connecting any two Ensembl IDs in *S*_oc_. From the obtained shortest paths, we extracted the inner nodes that did not represent validated OC-related genes. Genes corresponding to the extracted nodes were considered to be related to OC and denoted candidate genes. In addition, each candidate gene was assigned a measurement, called betweenness, which was defined as the number of shortest paths within which it was contained.

### Screening rules

The RWR and SP algorithms can produce a number of candidate genes for OC when the probability threshold is provided for the RWR algorithm. However, several false discoveries are inevitable, as mentioned in previous studies [33, 40]. To screen out these false discoveries, a series of screening rules were added to this section and integrated into the RWR-based and SP-based methods. Some parameters were employed to build the rules. Their optimal values in RWR-based and SP-based methods are determined by training these two methods, respectively.

The probability that a candidate gene produced by the RWR algorithm was influenced by the structure of the network *N*_RWR_ and the betweenness for a candidate gene produced by the SP algorithm was also clearly influenced by the structure of the *N*_SP_ network. Some candidate genes receiving high probabilities (betweenness) were not specific to OC. Thus, a permutation test was designed that first randomly constructed 1,000 sets of Ensembl IDs such that each set had the same size of *S*_oc_, denoted by *S*_1,_ *S*_2_,⋯, *S*_1000_. For each set, the RWR algorithm and SP algorithm were executed on *N*_RWR_ and *N*_SP_, respectively, by setting the Ensembl IDs in the set as the input, yielding a probability for each candidate gene produced by the RWR algorithm and a betweenness for each candidate gene produced by the SP algorithm. Finally, for each candidate gene, there was one probability (betweenness) for *S*_oc_ and 1,000 probabilities (betweenness) for *S*_1_, *S*_2_,⋯,*S*_1000_. If the candidate gene was specific to OC, its probability (betweenness) for *S*_oc_ should clearly be larger than most probabilities (betweenness) for *S*_1_, *S*_2_,⋯,*S*_1000_. Thus, we calculated the p-value for each candidate gene *g*, which was defined by

$$\text{p}-\text{value}(g)=\frac{\sum_{i=1}^{1000}\delta_{i}}{1000}$$ (3)

where *δ*_*i*_ = 1 if the probability (betweenness) for *S*_*i*_ was larger than that of *S*_oc_; *δ*_*i*_ = 0 otherwise. Because 0.05 was always used as an important cut-off for the significance level of the test, it was set to be the threshold of the p-value; *i*.*e*., candidate genes with p-values less than 0.05 were selected.

The network-based method is useful for complicated problems. However, this type of method seldom considers the essential properties of nodes in the network, leading to several false discoveries. Rules utilizing the essential properties of candidate and validated genes are needed to exclude false discoveries and select important ones.

As mentioned in the Section “Protein-protein interactions”, two proteins that can interact with each other always have a functional relationship. Furthermore, considering the interaction scores, proteins in an interaction with a high score are more likely to have a strong functional relationship than those in an interaction with a low score. Thus, the interaction score can be used to measure the associations between candidate genes and OC-related genes. For each candidate gene *g*, we calculated the maximum interaction score (*MIS*), which was defined by

$$MIS(g)=\text{max}\{S(g,g\prime ):g\prime \in S_{\text{oc}}\}$$ (4)

Clearly, a candidate gene with a high *MIS* is more likely to be a novel OC-related gene and thus should be selected. However, the threshold of *MIS* was not easy to determine. Thus, it was set as a parameter, *p*_*MIS*_. In the RWR-based method, the optimal value of *p*_*MIS*_ is determined by training this method. In addition, the optimal value of *p*_*MIS*_ in the SP-based method is also accessed by training the SP-based method.

The *MIS* measures the associations between candidate genes and OC-related genes. The following measurement evaluates the associations between them in another way. It is known that OC-related genes must be highly related to some gene ontology (GO) terms or biological pathways. Thus, we can use the annotated GO term and KEGG pathway information for candidate genes and OC-related genes to evaluate their correlations. To achieve this goal, each candidate gene or OC-related gene was encoded by its GO enrichment scores and KEGG enrichment scores. For a candidate gene *g* and an OC-related gene *g*′, their associations can be measured by

$$M(g,g\prime )=\frac{ES(g)\cdot ES(g\prime )}{\left\|ES(g)\right\|\cdot \left\|ES(g\prime )\right\|}$$ (5)

where *ES*(*g*) (*ES*(*g*′), respectively) is a vector consisting of the GO enrichment scores and KEGG enrichment scores of *g* (*g*′, respectively). A high value for Eq 5 indicated a strong association. Similar to the definition of *MIS*, we calculated the maximum function score (*MFS*) of each candidate gene *g* by

$$MFS(g)=\text{max}\{M(g,g\prime ):g\prime \in S_{\text{oc}}\}$$ (6)

Similarly, a candidate gene with a high *MFS* might be a novel OC-related gene with a high probability and should be selected. Additionally, it was difficult to determine the threshold of *MFS*. The parameter *p*_*MFS*_ was also established for this threshold. Similar to parameter *p*_*MIS*_ mentioned above, the optimal value of *p*_*MFS*_ in the RWR-based and SP-based methods is determined by training these two methods, respectively.

### Integrated method

The RWR-based and SP-based methods, together with the screen rules mentioned in the Section “Screening rules”, were constructed. The pseudo-codes of these two methods are listed in Tables 1 and 2, respectively. The integrated method encompassed these two methods by combining their results.

**Table 1:** The pseudo-code of the RWR-based method.

RWR-based method
**Input:** An OC-related gene set, *S*_oc_; a network, *N*_RWR_
**Output:** A number of putative OC-related genes
1. Execute the RWR algorithm on *N*_RWR_ using S_oc_ as the input, producing a probability for each gene in *N*_RWR_; select candidate genes with a probability higher than $p_{RWR}^{p}$;
2. Execute the permutation test, producing the p-value for each gene; select candidate genes with a p-value less than 0.05;
3. For each candidate gene, calculate its *MIS* and select candidate genes with an *MIS* no less than *p*_*MIS*_;
4. For each candidate gene, calculate its *MFS* and select candidate genes with an *MFS* larger than *p*_*MFS*_;
5. Output the remaining candidate genes as the putative OC-related genes.

**Table 2:** The pseudo-code of the SP-based method.

SP-based method
**Input:** An OC-related gene set, *S*_oc_; a network, *N*_SP_
**Output:** A number of putative OC-related genes
1. Execute the SP algorithm on *N*_SP_ using S_oc_ as the input, extracting candidate genes lying on the obtained shortest paths;
2. Execute the permutation test, producing the p-value for each candidate gene; select candidate genes with p-values less than 0.05;
3. For each candidate gene, calculate its *MIS* and select candidate genes with an *MIS* no less than *p*_*MIS*_;
4. For each candidate gene, calculate its *MFS* and select candidate genes with an *MFS* larger than *p*_*MFS*_;
5. Output the remaining candidate genes as the putative OC-related genes.

### Evaluation methods

The RWR-based and SP-based methods were applied to identify novel OC-related genes. However, there are some parameters in these two methods that must be determined before they are used to identify novel OC-related genes. Thus, these two methods were trained on the validated OC-related gene set *S*_oc_, through which the optimal parameters can be determined. We used the jackknife test [46, 47], which is one of the classic cross-validation methods [48, 49], to evaluate the performance of these two methods, *i*.*e*., each OC-related gene in *S*_oc_ was singled out sequentially, and the remaining genes in *S*_oc_ were used to generate predictions under various combinations of parameters. When training the methods, we supposed that all genes in the network other than the validated OC-related genes were negative; *i*.*e*., they were not OC-related genes. The performance of the method can be measured according to the following two features: (1) whether the selected OC-related gene can be recovered by executing the method on the remaining OC-related genes; (2) the predicted genes other than the selected OC-related gene should be as low as possible. Thus, we considered the following two measurements: precision and recall, which are always used to evaluate the performance of the methods on a binary classification problem in the fields of pattern recognition and information retrieval. Recall is defined as the proportion of retrieved OC-related genes among all OC-related genes, and precision represents the proportion of retrieved OC-related genes among all retrieved genes. Furthermore, another measurement, the F1-measure, is often used to evaluate overall performance, and it can be calculated by

$$F1-measure=\frac{2\cdot recall\cdot precision}{recall+precision}$$ (7)

Eq 7 shows that recall and precision have the same role. However, in this study, we considered recall to be more important than precision because the method with low recall and high precision could not reliably produce the predicted results. Thus, we simply revised Eq 7 and defined a new measurement, namely, F1-measure-R, which can be computed by

$$F1-measure-R=recall\cdot F1-measure=\frac{2\cdot recall^{2}\cdot precision}{recall+precision}$$ (8)

Because the retrieved genes differ when the selected OC-related gene was not the same, the recall, precision and F1-measure-R must be considered each time for the predicted results. Thus, under a combination of parameters, the RWR-based and SP-based methods can produce a series of recall, precision and F1-measure-R values. We calculated the average values to indicate the performance of the method under this combination of parameters. For convenience, the recall, precision and F1-measure-R presented in the rest of this paper represent the average values.

## Results

### Optimized parameters for the RWR-based and SP-based methods

As mentioned in the Section “RWR-based method”, “SP-based method” and “Screening rule”, some parameters should be optimized for the RWR-based method and the SP-base method. To extract an optimal combination of parameters for each method, the RWR-based method and the SP-base method were trained on *S*_oc_, and their performance was evaluated by the Jackknife test.

**Fig 1:**
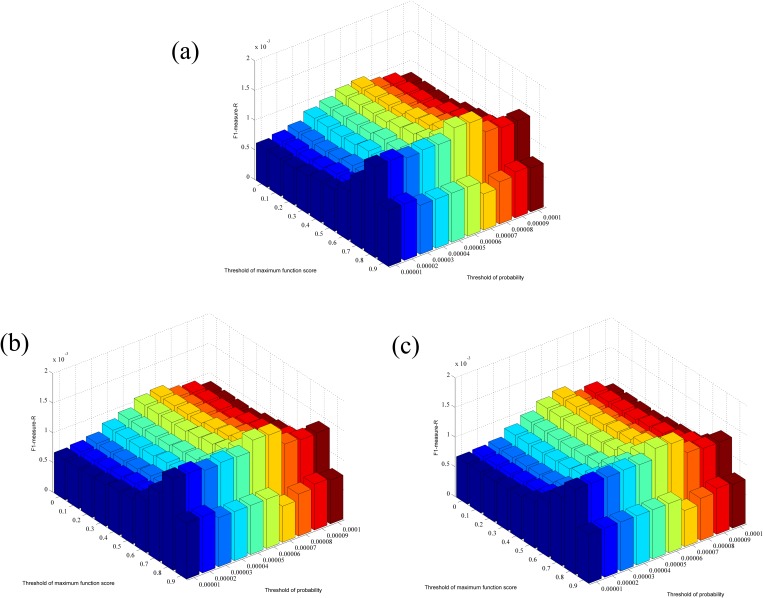
The performance of the RWR-based method under different combinations of parameters. (a) The performance of the RWR-based method setting *p*_*MIS*_ = 400. (b) The performance of the RWR-based method setting *p*_*MIS*_ = 700. (c) The performance of the RWR-based method setting *p*_*MIS*_ = 900.

For the RWR-based method, three parameters, the threshold of probability $p_{RWR}^{p}$, the threshold of MIS *p*_*MIS*_, and the threshold of MFS *p*_*MFS*_, should be optimized. For $p_{RWR}^{p}$, we tried various values ranging from 1E-05 to 1E-04; for *p*_*MIS*_, we tried three values, 400, 700, 900, which are reported in STRING for thresholds of medium confidence, high confidence and highest confidence, respectively; for *p*_*MFS*_, we tried various values ranging from 0 to 0.9. The measurements mentioned in the Section “Evaluation methods” for the RWR-based method with different combinations of parameters are listed in S2 Table. For ease of observation, the values of F1-measure-R obtained using the RWR-based method with different parameters are illustrated in Fig 1; we can see the same level of performance when the parameters $p_{RWR}^{p}$ and *p*_*MFS*_ are equivalent. The maximum F1-measure-R was 1.677E-03 when the parameters were set to be $p_{RWR}^{p}=0.00006$, *p*_*MFS*_ = 0.8 and *p*_*MIS*_ = 400 or 700. Because this combination of parameters yielded the best performance, they were used to build the optimal RWR-based method, which is adopted to identify novel OC-related genes.

**Fig 2:**
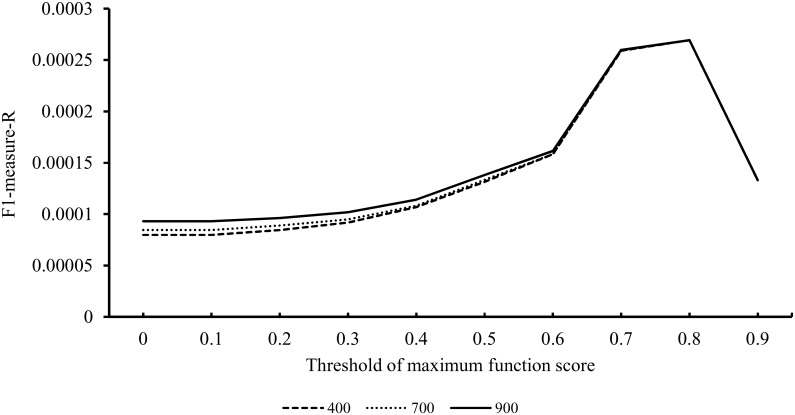
The performance of the SP-based method under different combinations of parameters. There are three lines in this figure, which represent the performance of the SP-based method with different thresholds of maximum interaction score. In detail, the full line represents the performance of the SP-based method with the threshold of maximum interaction score 900, the dot line represents the performance of the SP-based method with the threshold of maximum interaction score 700, the dash line represents the performance of the SP-based method with the threshold of maximum interaction score 400.

The SP-based method was also trained to extract the optimal parameters for *p*_*MIS*_ and *p*_*MFS*_. We tested three values for *p*_*MIS*_, as mentioned in the above paragraph, and various values ranging from 0 to 0.9 for *p*_*MFS*_. For the results obtained using the SP-based method with different combinations of parameters, the measurements mentioned in the Section “Evaluation methods” were counted and are provided in S3 Table. Additionally, three curves are plotted in Fig 2 to show the values of F1-measure-R obtained using the SP-based method with different values of *p*_*MFS*_ and a fixed value of *p*_*MIS*_. When the values of *p*_*MFS*_ were small, the values of F1-measure-R were proportional to the value of *p*_*MIS*_. However, the values of F1-measure-R were almost the same when *p*_*MFS*_ were large. The maximum F1-measure-R was 2.693E-04 when the parameters were set to be *p*_*MFS*_ = 0.8 and *p*_*MIS*_ = 400 or 700 or 900. Similarly, we used these values to build the optimal SP-based method for the identification of novel OC-related genes.

### Inferred results of the RWR-based method

As mentioned in the Section “Optimized parameters for the RWR-based and SP-based methods”, the optimal RWR-based method was built using $p_{RWR}^{p}=0.00006$, *p*_*MFS*_ = 0.8 and *p*_*MIS*_ = 400 or 700. This method was further used to identify novel OC-related genes based on all validated OC-related genes mentioned in the Section “Genes related to oral cancer”. Because the optimal RWR-based method contains two options for the parameter *p*_*MIS*_, this method was executed twice. First, *p*_*MIS*_ was set to 400, while in the second iteration, it was set to 700. The genes identified using this method with a different parameter, *p*_*MIS*_, are listed in S4 Table. A careful review of the results showed that the identified genes produced using these two optimal RWR-based methods were equivalent. They all identified 81 novel genes. Because all of these genes were viewed as high probability by the RWR method and had strong associations with validated OC-related genes, we believe that they are highly related to OC, and they are termed putative OC-related genes.

### Inferred results of the SP-based method

The optimal SP-based method was built in the Section “Optimized parameters for the RWR-based and SP-based methods”, in which *p*_*MFS*_ was set to 0.8 and *p*_*MIS*_ was set to 400, 700 or 900. This method was also adopted to identify novel OC-related genes. By setting *p*_*MIS*_ to 400, 700 or 900, we can build three optimal SP-based methods. They were executed sequentially to identify novel OC-related genes. Similarly, they all yielded fifteen identical genes, which are listed in Table 3. It is believed that these genes are closely related to OC, and they are termed putative OC-related genes.

**Table 3:** Genes identified by the optimal SP-based method.

Ensembl ID	Gene symbol	Betweenness	P-value	*MIS*	*MFS*	Function
ENSP00000354394	STAT1 ^b^	1443	<0.001	999	0.852	functions as a key factor in cell viability in response to different cell stimuli and pathogens [125]
ENSP00000263341	IL1B ^b^	543	<0.001	994	0.873	a member of the interleukin 1 cytokine family
ENSP00000379625	MYD88 ^a^	528	0.006	999	0.880	an essential signal transducer in the IL1 and Toll-like receptor signaling pathways [126, 127]
ENSP00000233946	IL1R1 ^b^	528	0.001	920	0.843	interleukin 1 receptor type 1 [128]
ENSP00000216797	NFKBIA ^b^	201	0.018	999	0.825	a member of the NF-kappa-B inhibitor family, which is involved in inflammatory responses [61]
ENSP00000222382	CYP3A43 ^b^	183	<0.001	958	0.988	a member of the cytochrome P450 superfamily of enzymes [129]
ENSP00000328181	NOG ^b^	183	0.005	999	0.862	binds and inactivates members of the TGF-beta superfamily signaling proteins [130]
ENSP00000410294	FGFR2 ^a^	183	0.01	999	0.846	a tyrosine protein kinase that functions as a receptor for fibroblast growth factors and plays key roles in cell proliferation, differentiation, migration and apoptosis [131]
ENSP00000362795	CXCR3 ^a^	179	0.021	999	0.808	a G protein-coupled receptor with selectivity for chemokines [132, 133]
ENSP00000260356	THBS1 ^a^	10	0.033	984	0.807	an adhesive glycoprotein that mediates cell-cell and cell-matrix interactions [134, 135]
ENSP00000264156	MCM6 ^b^	8	0.014	999	0.822	be involved in the formation of replication forks [136]
ENSP00000301141	CYP2A6 ^b^	3	0.016	950	0.948	a member of the cytochrome P450 superfamily of enzymes [129]
ENSP00000331736	SELE ^b^	1	0.006	978	0.852	responsible for the accumulation of blood leukocytes at sites of inflammation [67]
ENSP00000168712	FGF4 ^b^	1	0.016	999	0.847	fibroblast growth factor 4 which are involved in various biological processes such as cell growth and morphogenesis
ENSP00000286758	CXCL13 ^b^	1	0.005	992	0.838	C-X-C motif chemokine ligand 13

^a^: Genes that have shown stimulative or suppressive effects on cancer as validated by experiments.

^b^: Genes that have been reported to have a certain relationship with cancer but that have not been validated by experiments.

### Inferred results of the integrated method

**Fig 3:**
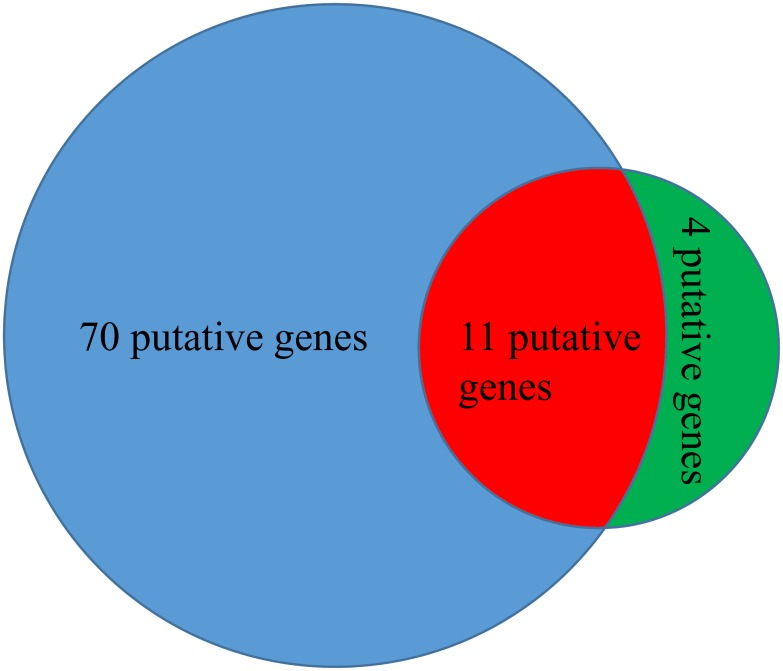
The distribution of the 85 putative OC-related genes obtained in this study. The blue part represents the set consisting of 70 putative genes obtained using only the RWR-based method. The red part represents the set consisting of 11 putative genes obtained using both the RWR-based and SP-based methods. The green part represents the set consisting of 4 putative genes obtained using only the SP-based method.

**Fig 4:**
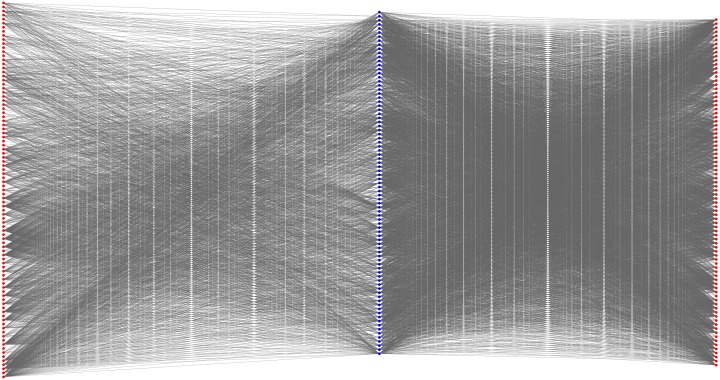
The sub-network containing the putative genes and OC-related genes that was extracted from the network for RWR-based and SP-based methods. The blue nodes represent putative genes and red nodes represent OC-related genes.

As mentioned in the Sections “Inferred results of the RWR-based method” and “Inferred results of the SP-based method”, the RWR-based method yielded 81 putative genes, and the SP-based method yielded fifteen putative genes. The union of these two putative gene sets provided the results of the integrated method, in which 85 putative genes were obtained. Their distribution is illustrated in Fig 3; we can see that eleven putative genes were identified by both the RWR-based and the SP-based methods. Among the remaining putative genes, 70 were identified using the RWR-based method, and four putative genes were identified using the SP-based method. Because the principles of the RWR-based and SP-based methods are very different, the identified novel OC-related genes were not the same. By considering the identified genes produced by either of them, we can obtain more putative genes and have an opportunity to extensively study disease genes in OC. To indicate the obtained 85 putative genes are highly related to OC, we extracted a sub-network containing these putative genes and OC-related genes from *N*_RWR_ and *N*_SP_ as shown in Fig 4.

## Discussion

Using the RWR-based method, 81 genes were obtained. Using the SP-based method, fifteen genes were accessed. All of these genes were deemed to be significantly associated with OC. Furthermore, eleven genes were identified by both methods, which may be more important than the others. In this section, some important putative genes (listed in Tables 4–6) were extensively analyzed to confirm their associations with OC.

**Table 4:** Eleven putative genes identified using both RWR-based and SP-based methods.

Ensembl ID	Gene symbol	RWR-based method				SP-based method				Function
		Probability	P-value	*MIS*	*MFS*	Betweenness	P-value	*MIS*	*MFS*	
ENSP00000379625	MYD88 ^a^	6.67E-05	0.032	999	0.880	528	0.006	999	0.880	an essential signal transducer in the IL1 and Toll-like receptor signaling pathways [126, 127]
ENSP00000410294	FGFR2 ^a^	9.04E-05	0.021	999	0.846	183	0.01	999	0.846	a tyrosine protein kinase that functions as a receptor for fibroblast growth factors and plays key roles in cell proliferation, differentiation, migration and apoptosis [131]
ENSP00000216797	NFKBIA ^b^	7.56E-05	0.026	999	0.825	201	0.018	999	0.825	a member of the NF-kappa-B inhibitor family, which is involved in inflammatory responses [61]
ENSP00000331736	SELE ^b^	9.73E-05	<0.001	978	0.852	1	0.006	978	0.852	responsible for the accumulation of blood leukocytes at sites of inflammation [67]
ENSP00000260356	THBS1 ^a^	6.94E-05	<0.001	984	0.807	10	0.033	984	0.807	an adhesive glycoprotein that mediates cell-cell and cell-matrix interactions [134, 135]
ENSP00000354394	STAT1 ^b^	4.36E-04	<0.001	999	0.852	1443	<0.001	999	0.852	functions as a key factor in cell viability in response to different cell stimuli and pathogens [125]
ENSP00000301141	CYP2A6 ^b^	8.85E-05	<0.001	950	0.948	3	0.016	950	0.948	a member of the cytochrome P450 superfamily of enzymes[129]
ENSP00000222382	CYP3A43 ^b^	3.35E-04	<0.001	958	0.988	183	<0.001	958	0.988	a member of the cytochrome P450 superfamily of enzymes [129]
ENSP00000286758	CXCL13 ^b^	6.63E-05	0.006	992	0.837	1	0.005	992	0.838	C-X-C motif chemokine ligand 13
ENSP00000168712	FGF4 ^b^	8.21E-05	0.001	999	0.847	1	0.016	999	0.847	fibroblast growth factor 4 which are involved in various biological processes such as cell growth and morphogenesis
ENSP00000263341	IL1B ^b^	1.92E-04	<0.001	994	0.873	543	<0.001	994	0.873	a member of the interleukin 1 cytokine family

^a^: Genes that have shown stimulative or suppressive effects on cancer as validated by experiments.

^b^: Genes that have been reported to have a certain relationship with cancer but that have not been validated by experiments.

**Table 5:** Important genes among the seventy putative genes identified using the RWR-based method.

Ensembl ID	Gene symbol	Probability	P-value	*MIS*	*MFS*	Function
ENSP00000245451	BMP4 ^a^	9.57E-05	0.026	981	0.905	bind TGF-beta receptor leading to recruitment and activation of transcription factor [137]
ENSP00000225831	CCL2 ^b^	1.21E-04	0.012	984	0.869	C-C motif chemokine ligand 2
ENSP00000351671	CCL20 ^b^	6.69E-05	0.003	965	0.804	C-C motif chemokine ligand 20
ENSP00000293272	CCL5 ^a^	7.70E-05	0.002	994	0.891	C-C motif chemokine ligand 5
ENSP00000292303	CCR5 ^a^	1.01E-04	0.003	996	0.839	C-C motif chemokine receptor 5
ENSP00000246657	CCR7 ^a^	9.82E-05	<0.001	998	0.823	C-C motif chemokine receptor 7
ENSP00000229135	IFNG ^a^	1.70E-04	0.03	994	0.839	binds to the interferon gamma receptor to response to infection [138]
ENSP00000228280	KITLG ^a^	1.05E-04	0.026	948	0.816	the ligand of the tyrosine-kinase receptor
ENSP00000162749	TNFRSF1A ^b^	9.69E-05	0.013	999	0.825	a member of the TNF receptor superfamily which plays a role in various biological processes
ENSP00000289153	PIK3CB ^a^	1.23E-04	<0.001	997	0.926	an isoform of the catalytic subunit of PI3K
ENSP00000366563	PIK3CD ^b^	1.15E-04	<0.001	997	0.919	PI3Ks phosphorylate inositol lipids and it is involved in the immune response [139]
ENSP00000352121	PIK3CG ^a^	1.21E-04	<0.001	996	0.921	phosphatidylinositol-4,5-bisphosphate 3-kinase catalytic subunit gamma
ENSP00000324648	CYP2B6 ^b^	9.73E-05	<0.001	962	0.943	a member of the cytochrome P450 superfamily
ENSP00000360372	CYP2C19 ^b^	9.32E-05	<0.001	962	0.966	cytochrome P450 family 2 subfamily C member 19
ENSP00000360247	CYP2J2 ^a^	7.48E-05	0.004	912	0.969	cytochrome P450 family 2 subfamily J member 2
ENSP00000337915	CYP3A4 ^b^	3.32E-04	<0.001	963	0.941	cytochrome P450 family 3 subfamily A member 4
ENSP00000360968	CYP4X1 ^b^	7.43E-05	0.018	939	0.959	cytochrome P450 family 4 subfamily X member 1
ENSP00000304283	RAC3 ^a^	1.08E-04	0.006	990	0.981	a GTPase regulates cell growth, cytoskeletal reorganization, and the activation of kinases [140–142]

^a^: Genes that have shown stimulative or suppressive effects on cancer as validated by experiments.

^b^: Genes that have been reported to have a certain relationship with cancer but that have not been validated by experiments.

**Table 6:** Four putative genes identified using the SP-based method.

Ensembl ID	Gene symbol	Betweenness	P-value	*MIS*	*MFS*	Function
ENSP00000264156	MCM6 ^b^	8	0.014	999	0.822	be involved in the formation of replication forks [136]
ENSP00000328181	NOG ^b^	183	0.005	999	0.862	binds and inactivates members of the TGF-beta superfamily signaling proteins [130]
ENSP00000362795	CXCR3 ^a^	179	0.021	999	0.808	a G protein-coupled receptor with selectivity for chemokines [132, 133]
ENSP00000233946	IL1R1 ^b^	528	0.001	920	0.843	interleukin 1 receptor type 1 [128]

^a^: Genes that have shown stimulative or suppressive effects on cancer as validated by experiments.

^b^: Genes that have been reported to have a certain relationship with cancer but that have not been validated by experiments.

### Putative genes identified using both RWR-based and SP-based methods

The following eleven proteins were identified using both RWR-based and SP-based methods: CYP3A43, FGF4, NFKBIA, THBS1, IL1B, CXCL13, CYP2A6, SELE, STAT1, MYD88 and FGFR2, as listed in Table 4.

MYD88 (myeloid differentiation primary response 88) encodes a cytosolic adapter protein that functions as a key signal transducer in the interleukin-1 and Toll-like receptor (TLRs) signaling pathways. TLRs and their ligands play a crucial role in inflammation and host defense [50]. In a broad variety of tumor tissues and cell lines, aberrant expression of TLRs plays a role in tumor immune escape or resistance to apoptosis [51, 52]. The expression of TLR4 and MyD88 is also aberrant and affects the down-stream signaling pathway [53]. In this study, MyD88 was predicted using both RWR-based and SP-based methods, and thus it may be a potential target for OC.

FGFR2 (fibroblast growth factor receptor 2) belongs to the FGFR tyrosine kinase family, which is one of the most frequently altered kinase families in some types of cancer [54]. Point mutations have been observed in 12% of endometrial carcinomas [55]. These studies suggest that FGFR2 may act together with the regulation of the PI3K/AKT/mTOR pathway to drive endometrial cancer growth in a subset of patients [56, 57]. FGFR2 mutations also occur in oral squamous cell carcinoma, and a patient with OSCC was found to respond to pazopanib, a multiple tyrosine kinase inhibitor [58]. These findings support the potential diagnostic and therapeutic value of FGFR2 as an effective strategy for the treatment of patients with OC with defined molecular characteristics. In addition, our results showed that FGF4 had a significant probability in both methods. Some large-scale experiments have suggested that aberrant amplification of FGF4 occurs in several types of cancer, including lymph node metastasis and urinary bladder cancer [59, 60].

NF-κBIA (nuclear factor-kappa B inhibitor alpha) inhibits NF-κB, which is involved in inflammatory responses and is a hallmark linking inflammation to tumor development and progression [61, 62]. The polymorphic variations in NFKBIA were associated with the risk of various cancers, including gastric cancer, prostate cancer and melanoma [63–65]. NFKBIA polymorphisms have significant associations with OSCC [66]. In our study, NFKBIA had a close relationship with OC using both methods.

SELE (selectin E), which was found in cytokine-stimulated endothelial cells, functions in the accumulation of blood leukocytes at sites of inflammation [67]. The level of SELE-mediated adhesion of colon cancer and head and neck squamous cell cancer cells to the endothelium has been implied to be involved in metastasis [68, 69]. Our analysis revealed that SELE might be a putative marker for tumorigenesis in OC. A study in Taiwan supports the idea that SELE-related inflammation plays a crucial role in the pathogenesis process of OSCC [70]. Future investigations of the function of SELE may clarify the mechanism of tumorigenesis and metastasis.

THBS1 (thrombospondin 1) is a subunit of a disulfide-linked homotrimeric protein, which mediates cell-cell and cell-matrix interactions [71]. The significant relationship between THBS1 and OC was detected using both RWR-based and SP-based methods. Several studies have indicated that THBS1 has crucial functions in oral tumorigenesis [72, 73]. THBS1 might be a potential diagnostic and therapeutic target for OC.

STAT1 (signal transducer and activator of transcription 1), which belongs to the STAT protein family, mediates the expression of a variety of genes and cell viability in response to stimuli and pathogens [74]. Aberrant activation of STAT1 has frequently been found in various cancers, such as head and neck cancer [75]. A similar STAT1 activation status was detected in patients with OSCC [76]. In this study, STAT1 displayed a significant association with OC using both the RWR-based and SP-based methods, and this molecular marker could help in guiding diagnostic and therapeutic decisions in patients with OC.

Among these eleven putative genes, including genes such as MYD88, FGFR2 and THBS1, stimulative or suppressive effects on cancers have been shown by the experiments. We speculate that these three genes have certain functions in OC. In addition, it has been reported that other genes, including NFKBIA, SELE, STAT1, IL1B, CYP2A6, CYP3A43, FGF4 and CXCL13 [77–80], have a certain relationship with various forms cancer. However, the mechanism has not been thoroughly studied. In our analysis, they have a significant relationship with OC, and the mechanism should be explored.

### Putative genes identified only by the RWR-based method

Seventy proteins were identified only by the RWR-based method. The important genes are BMP4, CCL2, CCL20, CCL5, CCR7, IFNG, KITLG, TNFRSF1A, PIK3CB, PIK3CD, PIK3CG, CYP2B6, CYP2C19, CYP2J2, CYP3A4, CYP4X1 and RAC3; these genes are listed in Table 5. Some of them participate in tumorigenesis by regulating cell growth or apoptosis, such as BMP4 and RAC3. Some genes are important factors in the immune system of cancer patients, such as CCL2, CCL20, CCL5, CCR7, IFNG and P450 family. Detailed function analyses of candidate genes are shown in S1 File.

We identified several novel putative genes using only the RWR-based method, which have strong associations with the tumorigenesis of OC. The functions of some genes have been explored in experiments, such as BMP4, IFNG, KITLG, CCL5, CCR5, CCR7, CYP2J2, PIK3CB, PIK3CG and RAC3; others revealed mutations or aberrant expression in cancers, but the mechanism is not clear. Future research is required to replicate and validate the effects of these genes.

### Putative genes identified only by the SP-based method

Four proteins were predicted to be closely associated with OC using the SP-based method but not the RWR-based method. These genes were IL1R1, MCM6, NOG and CXCR3; they are listed in Table 6. It has been reported that CXCR3 can promote metastasis in some types of cancer [81–86]. Other genes showed aberrant expression or mutations in cancers, which merits attention. Detailed function analyses of candidate genes are shown in S1 File.

Based on the analyses of the novel genes obtained by the integrated method, we found that several genes have shown stimulative or suppressive effects on cancers by experiments or aberrant expression or mutations in cancers reported in published papers, implying the effectiveness of the integrated method. In addition, the integrated method can provide more potential disease genes for investigating OC because it combines the results yielded by the RWR-based and SP-based methods; i.e., simultaneous usage of the RWR-based and SP-based method can help us mine more information about disease genes in oral cancer. We believe that it is helpful to investigate various diseases using both the RWR-based and the SP-based methods.

### GO-term and pathway function enrichment analysis of putative genes using DAVID

By the integrated method, 85 putative genes were obtained. To uncover the biological meaning behind these genes, the functional annotation tool, the Database for Annotation, Visualization and Integrated Discovery (DAVID) [87], was adopted to analyze them. The obtained results are provided in S5 Table.

For the GO enrichment results yielded by DAVID (see S5 Table), we can see that seven-two biological process (BP) GO terms, fifteen molecular function (MF) GO terms and nine cellular component (CC) GO terms are with statistically significance (FDR<0.05). The biological process mostly focused on cell proliferation, apoptosis, transcription and signal transduction. Malignant proliferation and anti-apoptosis are the major characteristics of many cancers. In this study, it has been found many proliferation related genes such as EGF, IGF1 and CDKN1B. EGF (epidermal growth factor) and its receptor EGFR affect cellular processes like proliferation, motility and adhesion. EGF has a higher expression and is correlated with the progression of cancer such as breast cancer [88]. The circulating IGF1 (insulin-like growth factor-1) level is associated with the risk to develop breast cancer [89, 90]. CDKN1B functions as a key cell cycle gatekeeper to prevent or slow down cell division [91, 92]. Aberrant downregulating CDKN1B may promote the proliferation of multiple myeloma cells [93]. Un-controlled expanded number of cancers is determined not only by cell proliferation but also by the cell evading apoptosis. In our study, many genes are enriched in apoptosis process such as TP53, CASP3 and cmyc. The mutations of TP53 are the most common alteration in cancer and are related to cell apoptosis, cell cycle and malignancy [94–97]. TP53 could arrest cell cycle and induced cell apoptosis in response to DNA damage [98]. It has been reported that over-expression of c-Myc drives the level of BAX and other apoptosis-related genes and is involved in cell apoptosis [99–103]. CASPs is a kind of cysteine-dependent aspartate-specific proteases, and is associated with the initiation and execution of apoptosis. As an activate effector, CASP3 receives the apoptotic signals to perform the cell death process [104]. In our result, a series of genes were enriched in the GO term related apoptosis, which proved process of apoptosis is a key process in oral cancer. In addition, several other biological process GO terms were shown such as transcription and signal transduction. In proliferation, apoptosis and other processes, functional genes were selectively transcribed and expressed. For example, c-Myc is a transcription factor which could mediate a series of down-stream genes express to drive cellular proliferation and apoptosis [105]. It can be seen from S5 Table that putative genes were enriched in several CC GO terms and MF GO terms such as growth factor activity (MF), cytokine activity (MF), enzyme or protein binding (MF), extracellular region (CC), extracellular space (CC) and plasma membrane (CC). These results suggest that the functional activity and localization of the protein are directly or indirectly related to oral cancer.

The DAVID also produced the KEGG pathway enrichment on putative genes (see S5 Table), several pathways were highlighted such as TNF signaling pathway, PI3K-Akt pathway, Pap1 pathway, MAPK pathway, TLR pathway, NF-kB pathway, JaK-STAT, Ras pathway and cytokine-cytokine receptor interaction. TNF pathway could be activated by various signals to affect the immunity, cell growth, apoptosis and other biological behaviors of tumor cells [106–110]. Protein-protein interaction and pathways is a cross-talk network. TNF can also activates the NF-kB pathway. The NF-kB pathway is activated in various cancers [111, 112]. Activation of NF-kB can be inhibited by the blockade of PI3K/Akt and ERK pathway to suppress tumor metastasis [113]. It was reported that PI3K/AKT-NF-kB is an axis which promotes bone metastasis in prostate cancer [114]. The Jak/STAT pathway is critical in normal tissues and tumors and the Jak kinase family includes JAK1, JAK2, JAK3 and TYK2 [115]. Jak mediated STAT phosphorylation leads to their nuclear translocation. STAT molecules bind specific promoter of genes and result in the transcription in nucleus, which regulate the cell proliferation, differentiation and apoptosis [115, 116]. It was reported that over-activation of the JAK/STAT pathway is related to subsets of patients with certain solid tumors and chronic myeloid leukemia [117, 118]. MAPK pathway is induced by activation of TLR (toll like receptors) and NOD (nucleotide-binding oligomerization domain receptors), which initiates of inflammation and are involved in cancer proliferation and control [119]. Ras pathway is a highly conserved pathway in cell function, including cell proliferation, differentiation and signaling transduction. This pathway is commonly deregulated in cancers, making the components in the pathway as targets for therapeutic interventions [120]. In these pathways, several putative genes were enriched, which indicates these pathway and gene need more attention in tumorigenesis of oral cancer.

## Conclusions

In this study, we investigated genes related to oral cancer. Two popular algorithms, the random walk with a restart algorithm and the shortest path algorithm, which are often used to identify novel disease genes, were integrated with some further rules to build two computational methods. An integrated method was further built by combining these two methods. To access an optimal prediction method for the identification of genes related to oral cancer using the integrated method, these two methods were trained on validated genes. The optimal prediction method was further adopted to identify novel genes related to oral cancer. The results indicated the following facts: (1) The integrated method is effective for identification of disease genes of oral cancer; (2) Candidate genes produced by the integrated method provide an opportunity to achieve a more extensive investigation of oral cancer. We hope that the integrated method can be useful for identifying novel disease genes. In view of the utility of the RWR-based and SP-based methods for identification of disease genes, it is hopeful that these methods can be applied to investigate other problems, such as DNA-binding protein prediction [121], protein fold recognition [122, 123], detection of tubule boundary [124], *etc*.

## Supporting information

S1 File Detailed analysis of the putative genes obtained by RWR-based method or SP-based method. (DOCX)

S1 Table 202 genes related to oral cancer and their sources. (DOCX)

S2 Table The performance of the RWR-based method with different combinations of parameters. (DOCX)

S3 Table The performance of the SP-based method with different combinations of parameters. (DOCX)

S4 Table Genes identified by the optimal RWR-based method. (DOCX)

S5 Table The enrichment analysis of putative genes yielded by DAVID. (XLSX)
