# Supplementary material for: An integrated method for the identification of novel genes related to oral cancer
Source: PLoS One. 2017 Apr 6;12(4):e0175185. doi: 10.1371/journal.pone.0175185 (PMC5383255; doi:10.1371/journal.pone.0175185)
Supplement: S1 File — (DOCX) [file pone.0175185.s001.docx]

**S1 File.** Detailed analysis of the putative genes obtained by RWR-based method or SP-based method

We obtained 81 genes using the RWR-based method. Using the SP-based method, fifteen genes were accessed.

**1. Putative genes identified only by the RWR-based method**

Seventy proteins were identified only by the RWR-based method. This section provides a detailed analysis of some of these genes.

BMP4 (bone morphogenetic protein 4), which belongs to the BMP family, a part of the TGF-β (transforming growth factor β) superfamily, is involved in embryonic development, cellular growth, differentiation and tumorigenesis [1]. BMP4 plays opposing roles in tumorigenesis and metastasis. In hepatocellular, breast and gastric cancer, the process of tumorigenesis is inhibited by BMP4 [2-4]. In glioblastomas, cell depletion is induced and tumorigenesis is inhibited by BMP4 [5]. In contrast, EMT (epithelial mesenchymal transition) is induced by BMP4 in pancreatic cancer cells [6]. In mice, knockdown of BMP4 inhibits cancer cell growth, migration, and invasion and suppresses tumorigenesis and metastasis of lung cancer cells [7]. BMP4 promotes tumor cell growth in ovarian cancer [6]. In our study, BMP4 acted as a potential factor using the RWR-based method. We believe that BMP4 may be a suitable therapeutic target for the diagnosis and treatment of OC.

Persistent inflammation is a critical factor in tumor initiation and progression, such as leukocyte recruitment, tumor cell proliferation and survival, invasion and metastasis [8, 9]. Monocytes/macrophages can be recruited by chemokines, which are secreted by tumor or stromal cells [10]. MCP-1 (monocyte chemotactic protein-1, also known as CCL2, C-C motif chemokine ligand 2) and MIP-1α/CCL3 (macrophage inflammatory protein-1 α) are small chemotactic proteins that have been found in several tumor types and functions as a key regulator of tumorigenesis [11]. In OSCC, serum CCL2 is a good diagnostic marker, and the CCL2/CCL3 ratio is associated with OSCC progression [11]. CCL2 is a powerful chemoattractant of CCR2^+^ (chemokine (C-C motif) receptor 2) monocytes/macrophages. Previous studies have demonstrated that the CCL2-CCR2 axis plays important roles in NSCLC (non-small cell lung cancer), breast cancer, ovarian cancer and squamous cell cancer [12-15]. CCL3 can react with its receptors, CCR1 and CCR5, and the CCL3-CCR5 axis may induce colitis-associated carcinogenesis through fibroblast accumulation [16]. CCL5, which is secreted by Schwann and dorsal root ganglion cells, and its receptor CCR5 are predominantly expressed in tumor cells and are associated with the invasion and metastasis of various types of tumors [17, 18]. In breast cancer cells, migration and invasion can be promoted by exogenous CCL5 and inhibited by CCR5 antagonist [19]. The CCL5/CCR5 axis plays a critical role in SACC (salivary adenoid cystic carcinoma) and enhanced migration of oral cancer cells via high levels of MMP-9 [20]. A case-control study suggested that SNPs in CCL5-28 and -403 genes could increase the risk of OC, and the CCL5-28 CG and -403 TT genes had combinative effects increasing the risk but reducing the clinicopathological development of OC [21]. It has been reported that CCL20 may contribute to the oral immune response to bacterial infection and may be related to OSCC cell growth [22]. CCR7 mediates the TNF-α induced metastasis of gallbladder cancer though regulation of JNK-AP-1 and ERK1/2-AP-1 signaling pathway[23]. In addition, CD105 elicits oral cancer cell motility through CCL20 expression [24]. Using the RWR-based method, CCL2/5/7/20 and CCR5/7 were predicted to be potential factors in OC. These chemokines and their receptors can be powerful diagnostic targets of OC.

IFNG (interferon, gamma) is a soluble cytokine that belongs to the type II interferon class. The protein, which is secreted by innate and adaptive immune cells, binds to the interferon gamma receptor to trigger the immune response [25]. This gene is associated with several immune diseases and tumors. IFNG, as an important anti-proliferative cytokine, is involved in multiple pathways to check tumorigenesis [26]. Polymorphisms in IFNG and the interaction between IFNG and CD28 may contribute to susceptibility to cervical cancer [25, 27, 28]. IFNG production and immunomodulatory activity may be regulated by NFκB [29, 30]. In colorectal cancer patients, the expression of IFNG was suppressed in peripheral blood mononuclear cells [31]. According to the results obtained with the RWR-based method, IFNG could be an effective biological marker for OC.

KITLG (KIT ligand) is the ligand of tyrosine kinase receptor and is believed to participate in cell migration. KITLG triggers its receptor c-KIT to function as a stem cell, steel and mast cell growth factor [32-34]. Over-expression of KITLG contributes to the carcinogenesis of breast cancer, NSCLC, colorectal and uveal melanoma [35-39]. It has been speculated that KITLG enhances proliferation and invasion mainly through the PI3K/Akt pathway [40]. The mechanism of KITLG in tumorigenesis remains largely unexplored. Our results showed that KITLG had a highly significant relationship with OC and may be a putative biomarker and therapeutic target for this condition.

The cytochrome P450 enzyme superfamily, composed of eighteen distinct families, consists of constitutive and inducible mono-oxygenases [41-44]. Aberrant P450 expression has been detected in a range of tumor types and identified as potential targets [45-49]. Among the putative genes obtained using the RWR-based method, several CYP family members may play putative roles in OC, such as CYP2B6, CYP2C19, CYP2J2, CYP3A4 and CYP4X1. The members of this family, CYP3A43 and CYP2A6, were also predicted using both methods. CYP2C19 participates in the metabolism of chemotherapeutic drugs, including cyclophosphamide, thalidomide, bortezomib, and tamoxifen [50-53]. This function suggests that the CYP2C19 genotype is associated with susceptibility via different carcinogen detoxification abilities [54]. CYP2C19 might participate in and initiate procarcinogen activity, ultimately increasing the risk of breast, lung and gastric cancer [54-57]. In comparison to the non-cancer cell lines or adjacent normal tissues, the expression of CYP2J2 is elevated in diverse human-derived cancer cell lines and human cancer tissues [58]. Overexpression of CYP2J2 promotes tumor malignancy, including proliferation and metastasis via activation of the MAPK (mitogen-activated protein kinase) and PI3K (phosphoinositide 3-kinase)/Akt pathway, whereas these effects are attenuated by inhibition of CYP2J2 [58-62]. CYP3A4 and CYP3A43 belong to the CYP3A subfamily, and the CYP3A4 variant is associated with a higher PCa (prostate cancer) clinical grade and stage, especially in older patients [63, 64]. CYP4X1 is located on chromosome 1 and predominantly expressed in adult human skeletal muscle, trachea, and aorta [65]. The influence of these proteins in OC is unclear, and we speculate that these proteins may be useful for the drug design of OC.

TNF-α is an inflammatory cytokine that is produced by immune cells and interacts with its receptors, TNFRSF1A and TNFRSF1B, to participate in inflammation [66]. Our results revealed that TNFRSF1A could act as a key tumor mediator in OC. TNF-α executes its biologic function, such as destroying tumor blood vessels and inducing the apoptosis of tumor cells, by binding to TNFRSF1A or TNFRSF1B [66]. However, TNF-α can promote tumorigenesis by inducing DNA damage, enhancing pro-angiogenic effects, increasing the production of MMPs (matrix metalloproteinases) and inducing growth-promoting hormone in the presence of chronic inflammation in the tumor microenvironment [67-69]. In breast cancer cell lines, silencing TNFRSF1A or TNFRSF1B can impair tumor survival signaling and the function of TNF-α [70]. In addition, SNPs in TNFRSF1A and TNFFSF1B have been considered to be associated with the risk of sporadic breast cancer [71]. The influence of TNFRSF1A in OC is still unclear, and the results produced using the RWR-based method indicates that it may play crucial roles by interacting with TNF-α in OC.

PI3K (phosphatidylinositide 3-kinase) signaling is regarded as an important pathway in multiple biological function. According to its catalytic isoforms, PI3Ks are classified into PIK3CA, PIK3CB and PIK3CD, and class IB (PIK3CG) [72]. PI3K has been suggested to contribute to MM (multiple myeloma) cell survival and downstream signaling via the PI3K/Akt axis [73-78]. Aberrant PI3K is involved in the promotion of cell proliferation and migration and the inhibition of apoptosis in various types of cancers [72, 79-84]. Mutations in PIK3CD are related to primary immunodeficiency in patients [85, 86]. PIK3CD, which is activated by GPCRs (G-protein coupled receptors), and its function in cancer remain controversial [87, 88]. PIK3CG was identified as a direct transcriptional target of Notch signaling and promotes migration and tumor sphere formation in breast cancer [89]. However, the function of these proteins remains unexplored. In this study, PIK3CB, PIK3CD and PIK3DG were identified as potential key factors in OC.

RAC3 (ras-related C3 botulinum toxin substrate 3) belongs to the Rho GTPase (guanosine triphosphatases) family, which acts as molecular switches in a variety of biological response pathways such as the cell cycle, cell motility and cell transformation [90, 91]. RAC3 GTPase is an effector of Pak in human breast cancer-derived epithelial cell lines, and the both proteins are critical for DNA synthesis [92]. The RAC3-Paks pathway can regulate the function of retinoblastoma protein and the expression of cyclin D1 [93, 94]. Overexpression of RAC3 has been observed in several cancers [95, 96]. In lung cancer cells, silencing RAC3 reduces proliferation and induces G0/G1 phase arrest [97]. However, the role of RAC3 in OC is less well understood, and we postulate that RAC3 may be a potential target for OC.

**2. Putative genes identified only by the SP-based method**

Four proteins were predicted to be closely associated with OC using the SP-based method but not the RWR-based method. They were IL1R1, MCM6, NOG and CXCR3.

MCM (mini-chromosome maintenance) proteins are key proteins in the initiation of DNA synthesis and replication. This family has been shown to be associated with histological grades in various tumors, such as in the prognostic value of MCM2, MCM4 and MCM7 in lung cancer [98]. Recently, it has been reported that MCM6 is a potential prognostic marker in NSCLC [99-102]. To our knowledge, MCM6 has not yet been detected in OC. The betweenness of MCM6 produced by the SP-based method was 8, and the permutation FDR was less than 0.05 (0.014). There data indicated that MCM6 plays key roles in OC.

BMPs (bone morphogenetic proteins) are a type of proteins belonging to the TGF-β (transforming growth factor-β) superfamily [103]. NOG (Noggin) is an antagonist of BMPs that exerts its effects on the shape and size of BMP-derived structures [104]. In NSCLC, it has been indicated that noggin suppresses the oncogenic properties of tumor cells in vitro and decreases the tumor size in vivo [105]. In breast cancer, the expression of noggin provides the capability of bone colonization [106]. In prostate cancer, noggin is implicated as a potential therapeutic factor to ameliorate prostate bone metastases and attendant pain and morbidity [107]. Noggin had a betweenness of 183 and a permutation FDR of 0.005, demonstrating its close relationship with OC. Chemokine receptors are a family of G-protein-coupled receptors with selectivity for chemokines. Binding of chemokines to this protein induces integrin activation, cytoskeletal changes and chemotactic migration [108, 109]. In the present study, CXCL13 was identified using both RWR-based and SP-based methods. CXCL1, CXCL12, and CXCR3 have been demonstrated to have a very significant association with OC using the RWR-based and the SP-based methods. CXCR3 can promote metastasis in breast cancer, colon cancer, lung cancer and osteosarcoma [110-115]. Silencing of CXCR3 was found to inhibit the migration and metastasis of breast cancer cells. In tumor-bearing mice, targeting CXCR3 with AMG487 (a small molecular inhibitor) significantly suppressed tumor metastasis and promoted host anti-tumor immunity [116]. CXCR3 had a betweenness of 179 and a permutation FDR of less than 0.05 (0.021), indicating a significant relationship with OC.

In addition, IL1R1 had a betweenness of 528 and a significant permutation FDR of 0.001 in the SP-based method. Thus, it may have potential immunity effects in OC.

We found that the novel genes obtained using two methods have some complementarity. Their simultaneous usage can help us better understand the mechanism of oral cancer.

**References**

1. Kallioniemi A. Bone morphogenetic protein 4-a fascinating regulator of cancer cell behavior. Cancer genetics. 2012;205(6):267-77. doi: 10.1016/j.cancergen.2012.05.009. PubMed PMID: 22749032.

2. Lee YC, Cheng CJ, Bilen MA, Lu JF, Satcher RL, Yu-Lee LY, et al. BMP4 promotes prostate tumor growth in bone through osteogenesis. Cancer Res. 2011;71(15):5194-203. doi: 10.1158/0008-5472.CAN-10-4374. PubMed PMID: 21670081; PubMed Central PMCID: PMC3148283.

3. Cao Y, Slaney CY, Bidwell BN, Parker BS, Johnstone CN, Rautela J, et al. BMP4 inhibits breast cancer metastasis by blocking myeloid-derived suppressor cell activity. Cancer Res. 2014;74(18):5091-102. doi: 10.1158/0008-5472.CAN-13-3171. PubMed PMID: 25224959.

4. Shirai YT, Ehata S, Yashiro M, Yanagihara K, Hirakawa K, Miyazono K. Bone morphogenetic protein-2 and -4 play tumor suppressive roles in human diffuse-type gastric carcinoma. Am J Pathol. 2011;179(6):2920-30. doi: 10.1016/j.ajpath.2011.08.022. PubMed PMID: 21996676; PubMed Central PMCID: PMC3260801.

5. Piccirillo SG, Reynolds BA, Zanetti N, Lamorte G, Binda E, Broggi G, et al. Bone morphogenetic proteins inhibit the tumorigenic potential of human brain tumour-initiating cells. Nature. 2006;444(7120):761-5. Epub 2006/12/08. doi: nature05349 [pii]

10.1038/nature05349. PubMed PMID: 17151667.

6. Hamada S, Satoh K, Hirota M, Kimura K, Kanno A, Masamune A, et al. Bone morphogenetic protein 4 induces epithelial-mesenchymal transition through MSX2 induction on pancreatic cancer cell line. Journal of cellular physiology. 2007;213(3):768-74. doi: 10.1002/jcp.21148. PubMed PMID: 17516553.

7. Kim JS, Kurie JM, Ahn YH. BMP4 depletion by miR-200 inhibits tumorigenesis and metastasis of lung adenocarcinoma cells. Molecular cancer. 2015;14(1):173. doi: 10.1186/s12943-015-0441-y. PubMed PMID: 26395571; PubMed Central PMCID: PMC4580148.

8. Lee HW, Choi HJ, Ha SJ, Lee KT, Kwon YG. Recruitment of monocytes/macrophages in different tumor microenvironments. Biochim Biophys Acta. 2013;1835(2):170-9. doi: 10.1016/j.bbcan.2012.12.007. PubMed PMID: 23287570.

9. Galdiero MR, Bonavita E, Barajon I, Garlanda C, Mantovani A, Jaillon S. Tumor associated macrophages and neutrophils in cancer. Immunobiology. 2013;218(11):1402-10. doi: 10.1016/j.imbio.2013.06.003. PubMed PMID: 23891329.

10. Allavena P, Germano G, Marchesi F, Mantovani A. Chemokines in cancer related inflammation. Exp Cell Res. 2011;317(5):664-73. doi: 10.1016/j.yexcr.2010.11.013. PubMed PMID: 21134366.

11. Ding L, Li B, Zhao Y, Fu YF, Hu EL, Hu QG, et al. Serum CCL2 and CCL3 as potential biomarkers for the diagnosis of oral squamous cell carcinoma. Tumour Biol. 2014;35(10):10539-46. doi: 10.1007/s13277-014-2306-1. PubMed PMID: 25060177.

12. Ueno T, Toi M, Saji H, Muta M, Bando H, Kuroi K, et al. Significance of macrophage chemoattractant protein-1 in macrophage recruitment, angiogenesis, and survival in human breast cancer. Clin Cancer Res. 2000;6(8):3282-9. PubMed PMID: 10955814.

13. Arenberg DA, Keane MP, DiGiovine B, Kunkel SL, Strom SR, Burdick MD, et al. Macrophage infiltration in human non-small-cell lung cancer: the role of CC chemokines. Cancer immunology, immunotherapy : CII. 2000;49(2):63-70. PubMed PMID: 10823415.

14. Riethdorf L, Riethdorf S, Gutzlaff K, Prall F, Loning T. Differential expression of the monocyte chemoattractant protein-1 gene in human papillomavirus-16-infected squamous intraepithelial lesions and squamous cell carcinomas of the cervix uteri. Am J Pathol. 1996;149(5):1469-76. PubMed PMID: 8909236; PubMed Central PMCID: PMC1865287.

15. Negus RP, Stamp GW, Relf MG, Burke F, Malik ST, Bernasconi S, et al. The detection and localization of monocyte chemoattractant protein-1 (MCP-1) in human ovarian cancer. J Clin Invest. 1995;95(5):2391-6. doi: 10.1172/JCI117933. PubMed PMID: 7738202; PubMed Central PMCID: PMC295866.

16. Sasaki S, Baba T, Shinagawa K, Matsushima K, Mukaida N. Crucial involvement of the CCL3-CCR5 axis-mediated fibroblast accumulation in colitis-associated carcinogenesis in mice. International journal of cancer Journal international du cancer. 2014;135(6):1297-306. doi: 10.1002/ijc.28779. PubMed PMID: 24510316.

17. Vaday GG, Peehl DM, Kadam PA, Lawrence DM. Expression of CCL5 (RANTES) and CCR5 in prostate cancer. The Prostate. 2006;66(2):124-34. doi: 10.1002/pros.20306. PubMed PMID: 16161154.

18. Borczuk AC, Papanikolaou N, Toonkel RL, Sole M, Gorenstein LA, Ginsburg ME, et al. Lung adenocarcinoma invasion in TGFbetaRII-deficient cells is mediated by CCL5/RANTES. Oncogene. 2008;27(4):557-64. doi: 10.1038/sj.onc.1210662. PubMed PMID: 17653092; PubMed Central PMCID: PMC2796568.

19. Velasco-Velazquez M, Jiao X, De La Fuente M, Pestell TG, Ertel A, Lisanti MP, et al. CCR5 antagonist blocks metastasis of basal breast cancer cells. Cancer Res. 2012;72(15):3839-50. doi: 10.1158/0008-5472.CAN-11-3917. PubMed PMID: 22637726.

20. Shen Z, Li T, Chen D, Jia S, Yang X, Liang L, et al. The CCL5/CCR5 axis contributes to the perineural invasion of human salivary adenoid cystic carcinoma. Oncology reports. 2014;31(2):800-6. doi: 10.3892/or.2013.2920. PubMed PMID: 24337063.

21. Weng CJ, Chien MH, Lin CW, Chung TT, Zavras AI, Tsai CM, et al. Effect of CC chemokine ligand 5 and CC chemokine receptor 5 genes polymorphisms on the risk and clinicopathological development of oral cancer. Oral Oncol. 2010;46(10):767-72. doi: 10.1016/j.oraloncology.2010.07.011. PubMed PMID: 20729133.

22. Abiko Y, Nishimura M, Kusano K, Nakashima K, Okumura K, Arakawa T, et al. Expression of MIP-3alpha/CCL20, a macrophage inflammatory protein in oral squamous cell carcinoma. Arch Oral Biol. 2003;48(2):171-5. PubMed PMID: 12642237.

23. Hong H, He C, Zhu S, Zhang Y, Wang X, She F, et al. CCR7 mediates the TNF-alpha-induced lymphatic metastasis of gallbladder cancer through the "ERK1/2 - AP-1" and "JNK - AP-1" pathways. Journal of experimental & clinical cancer research : CR. 2016;35:51. doi: 10.1186/s13046-016-0318-y. PubMed PMID: 27009073; PubMed Central PMCID: PMC4806413.

24. Chen CH, Chuang HC, Lin YT, Fang FM, Huang CC, Chen CM, et al. Circulating CD105 shows significant impact in patients of oral cancer and promotes malignancy of cancer cells via CCL20. Tumour Biol. 2015. doi: 10.1007/s13277-015-3991-0. PubMed PMID: 26334621.

25. Guzman VB, Yambartsev A, Goncalves-Primo A, Silva ID, Carvalho CR, Ribalta JC, et al. New approach reveals CD28 and IFNG gene interaction in the susceptibility to cervical cancer. Hum Mol Genet. 2008;17(12):1838-44. doi: 10.1093/hmg/ddn077. PubMed PMID: 18337305; PubMed Central PMCID: PMC2536747.

26. Brandacher G, Winkler C, Schroecksnadel K, Margreiter R, Fuchs D. Antitumoral activity of interferon-gamma involved in impaired immune function in cancer patients. Curr Drug Metab. 2006;7(6):599-612. PubMed PMID: 16918315.

27. Calhoun ES, McGovern RM, Janney CA, Cerhan JR, Iturria SJ, Smith DI, et al. Host genetic polymorphism analysis in cervical cancer. Clin Chem. 2002;48(8):1218-24. PubMed PMID: 12142377.

28. Dybikowska A, Sliwinski W, Emerich J, Podhajska AJ. Evaluation of Fas gene promoter polymorphism in cervical cancer patients. Int J Mol Med. 2004;14(3):475-8. PubMed PMID: 15289903.

29. Rudd CE, Schneider H. Unifying concepts in CD28, ICOS and CTLA4 co-receptor signalling. Nat Rev Immunol. 2003;3(7):544-56. doi: 10.1038/nri1131. PubMed PMID: 12876557.

30. Wei L, Sandbulte MR, Thomas PG, Webby RJ, Homayouni R, Pfeffer LM. NFkappaB negatively regulates interferon-induced gene expression and anti-influenza activity. J Biol Chem. 2006;281(17):11678-84. doi: 10.1074/jbc.M513286200. PubMed PMID: 16517601; PubMed Central PMCID: PMC1457055.

31. Ganapathi SK, Beggs AD, Hodgson SV, Kumar D. Expression and DNA methylation of TNF, IFNG and FOXP3 in colorectal cancer and their prognostic significance. British journal of cancer. 2014;111(8):1581-9. doi: 10.1038/bjc.2014.477. PubMed PMID: 25225903; PubMed Central PMCID: PMC4200101.

32. Rovasio RA, Faas L, Battiato NL. Insights into stem cell factor chemotactic guidance of neural crest cells revealed by a real-time directionality-based assay. Eur J Cell Biol. 2012;91(5):375-90. doi: 10.1016/j.ejcb.2011.12.007. PubMed PMID: 22382085.

33. Miyoshi T, Otsuka F, Nakamura E, Inagaki K, Ogura-Ochi K, Tsukamoto N, et al. Regulatory role of kit ligand-c-kit interaction and oocyte factors in steroidogenesis by rat granulosa cells. Mol Cell Endocrinol. 2012;358(1):18-26. doi: 10.1016/j.mce.2012.02.011. PubMed PMID: 22366471.

34. Horvath VJ, Vittal H, Lorincz A, Chen H, Almeida-Porada G, Redelman D, et al. Reduced stem cell factor links smooth myopathy and loss of interstitial cells of cajal in murine diabetic gastroparesis. Gastroenterology. 2006;130(3):759-70. doi: 10.1053/j.gastro.2005.12.027. PubMed PMID: 16530517.

35. Han ZB, Ren H, Zhao H, Chi Y, Chen K, Zhou B, et al. Hypoxia-inducible factor (HIF)-1 alpha directly enhances the transcriptional activity of stem cell factor (SCF) in response to hypoxia and epidermal growth factor (EGF). Carcinogenesis. 2008;29(10):1853-61. doi: 10.1093/carcin/bgn066. PubMed PMID: 18339685.

36. Levina V, Marrangoni A, Wang T, Parikh S, Su Y, Herberman R, et al. Elimination of human lung cancer stem cells through targeting of the stem cell factor-c-kit autocrine signaling loop. Cancer Res. 2010;70(1):338-46. doi: 10.1158/0008-5472.CAN-09-1102. PubMed PMID: 20028869; PubMed Central PMCID: PMC4572892.

37. Sun L, Hui AM, Su Q, Vortmeyer A, Kotliarov Y, Pastorino S, et al. Neuronal and glioma-derived stem cell factor induces angiogenesis within the brain. Cancer Cell. 2006;9(4):287-300. doi: 10.1016/j.ccr.2006.03.003. PubMed PMID: 16616334.

38. Lefevre G, Glotin AL, Calipel A, Mouriaux F, Tran T, Kherrouche Z, et al. Roles of stem cell factor/c-Kit and effects of Glivec/STI571 in human uveal melanoma cell tumorigenesis. J Biol Chem. 2004;279(30):31769-79. doi: 10.1074/jbc.M403907200. PubMed PMID: 15145934.

39. Yang S, Li WS, Dong F, Sun HM, Wu B, Tan J, et al. KITLG is a novel target of miR-34c that is associated with the inhibition of growth and invasion in colorectal cancer cells. J Cell Mol Med. 2014;18(10):2092-102. doi: 10.1111/jcmm.12368. PubMed PMID: 25213795; PubMed Central PMCID: PMC4244023.

40. Yasuda A, Sawai H, Takahashi H, Ochi N, Matsuo Y, Funahashi H, et al. Stem cell factor/c-kit receptor signaling enhances the proliferation and invasion of colorectal cancer cells through the PI3K/Akt pathway. Digest Dis Sci. 2007;52(9):2292-300. doi: 10.1007/s10620-007-9759-7. PubMed PMID: 17410437.

41. Danielson PB. The cytochrome P450 superfamily: biochemistry, evolution and drug metabolism in humans. Curr Drug Metab. 2002;3(6):561-97. PubMed PMID: 12369887.

42. Nebert DW, Russell DW. Clinical importance of the cytochromes P450. Lancet. 2002;360(9340):1155-62. doi: 10.1016/S0140-6736(02)11203-7. PubMed PMID: 12387968.

43. Guengerich FP, Shimada T. Activation of procarcinogens by human cytochrome P450 enzymes. Mutat Res. 1998;400(1-2):201-13. PubMed PMID: 9685642.

44. Windmill KF, McKinnon RA, Zhu X, Gaedigk A, Grant DM, McManus ME. The role of xenobiotic metabolizing enzymes in arylamine toxicity and carcinogenesis: functional and localization studies. Mutat Res. 1997;376(1-2):153-60. PubMed PMID: 9202751.

45. Patterson LH, Murray GI. Tumour cytochrome P450 and drug activation. Curr Pharm Des. 2002;8(15):1335-47. PubMed PMID: 12052211.

46. Murray GI. The role of cytochrome P450 in tumour development and progression and its potential in therapy. The Journal of pathology. 2000;192(4):419-26. doi: 10.1002/1096-9896(2000)9999:9999<::AID-PATH750>3.0.CO;2-0. PubMed PMID: 11113857.

47. Murray GI, Taylor MC, McFadyen MC, McKay JA, Greenlee WF, Burke MD, et al. Tumor-specific expression of cytochrome P450 CYP1B1. Cancer Res. 1997;57(14):3026-31. PubMed PMID: 9230218.

48. Kapucuoglu N, Coban T, Raunio H, Pelkonen O, Edwards RJ, Boobis AR, et al. Expression of CYP3A4 in human breast tumour and non-tumour tissues. Cancer letters. 2003;202(1):17-23. PubMed PMID: 14643022.

49. Rooney PH, Telfer C, McFadyen MC, Melvin WT, Murray GI. The role of cytochrome P450 in cytotoxic bioactivation: future therapeutic directions. Current cancer drug targets. 2004;4(3):257-65. PubMed PMID: 15134533.

50. Helsby NA, Hui CY, Goldthorpe MA, Coller JK, Soh MC, Gow PJ, et al. The combined impact of CYP2C19 and CYP2B6 pharmacogenetics on cyclophosphamide bioactivation. Br J Clin Pharmacol. 2010;70(6):844-53. doi: 10.1111/j.1365-2125.2010.03789.x. PubMed PMID: 21175440; PubMed Central PMCID: PMC3014068.

51. Ando Y, Fuse E, Figg WD. Thalidomide metabolism by the CYP2C subfamily. Clin Cancer Res. 2002;8(6):1964-73. PubMed PMID: 12060642.

52. Uttamsingh V, Lu C, Miwa G, Gan LS. Relative contributions of the five major human cytochromes P450, 1A2, 2C9, 2C19, 2D6, and 3A4, to the hepatic metabolism of the proteasome inhibitor bortezomib. Drug metabolism and disposition: the biological fate of chemicals. 2005;33(11):1723-8. doi: 10.1124/dmd.105.005710. PubMed PMID: 16103134.

53. Schroth W, Antoniadou L, Fritz P, Schwab M, Muerdter T, Zanger UM, et al. Breast cancer treatment outcome with adjuvant tamoxifen relative to patient CYP2D6 and CYP2C19 genotypes. J Clin Oncol. 2007;25(33):5187-93. doi: 10.1200/JCO.2007.12.2705. PubMed PMID: 18024866.

54. Bai L, He J, He GH, He JC, Xu F, Xu GL. Association of CYP2C19 polymorphisms with survival of breast cancer patients using tamoxifen: results of a meta- analysis. Asian Pac J Cancer Prev. 2014;15(19):8331-5. PubMed PMID: 25339025.

55. Chen J, Zheng X, Liu DY, Zhao Q, Wu YW, Tan FL, et al. Therapeutic effects and adverse drug reactions are affected by icotinib exposure and CYP2C19 and EGFR genotypes in Chinese non-small cell lung cancer patients. Asian Pac J Cancer Prev. 2014;15(17):7195-200. PubMed PMID: 25227813.

56. Shi WX, Chen SQ. Frequencies of poor metabolizers of cytochrome P450 2C19 in esophagus cancer, stomach cancer, lung cancer and bladder cancer in Chinese population. World J Gastroenterol. 2004;10(13):1961-3. PubMed PMID: 15222046; PubMed Central PMCID: PMC4572240.

57. Sugimoto M, Furuta T, Shirai N, Nakamura A, Kajimura M, Sugimura H, et al. Poor metabolizer genotype status of CYP2C19 is a risk factor for developing gastric cancer in Japanese patients with Helicobacter pylori infection. Alimentary pharmacology & therapeutics. 2005;22(10):1033-40. doi: 10.1111/j.1365-2036.2005.02678.x. PubMed PMID: 16268979.

58. Jiang JG, Ning YG, Chen C, Ma D, Liu ZJ, Yang S, et al. Cytochrome p450 epoxygenase promotes human cancer metastasis. Cancer Res. 2007;67(14):6665-74. doi: 10.1158/0008-5472.CAN-06-3643. PubMed PMID: 17638876.

59. Jiang C, Guo J, Wang Z, Xiao B, Lee HJ, Lee EO, et al. Decursin and decursinol angelate inhibit estrogen-stimulated and estrogen-independent growth and survival of breast cancer cells. Breast cancer research : BCR. 2007;9(6):R77. doi: 10.1186/bcr1790. PubMed PMID: 17986353; PubMed Central PMCID: PMC2246173.

60. Jiang JG, Chen CL, Card JW, Yang S, Chen JX, Fu XN, et al. Cytochrome P450 2J2 promotes the neoplastic phenotype of carcinoma cells and is up-regulated in human tumors. Cancer Res. 2005;65(11):4707-15. doi: 10.1158/0008-5472.CAN-04-4173. PubMed PMID: 15930289.

61. Chen C, Li G, Liao W, Wu J, Liu L, Ma D, et al. Selective inhibitors of CYP2J2 related to terfenadine exhibit strong activity against human cancers in vitro and in vivo. The Journal of pharmacology and experimental therapeutics. 2009;329(3):908-18. doi: 10.1124/jpet.109.152017. PubMed PMID: 19289568; PubMed Central PMCID: PMC2683771.

62. Chen C, Wei X, Rao X, Wu J, Yang S, Chen F, et al. Cytochrome P450 2J2 is highly expressed in hematologic malignant diseases and promotes tumor cell growth. The Journal of pharmacology and experimental therapeutics. 2011;336(2):344-55. doi: 10.1124/jpet.110.174805. PubMed PMID: 21030485; PubMed Central PMCID: PMC3033713.

63. Paris PL, Kupelian PA, Hall JM, Williams TL, Levin H, Klein EA, et al. Association between a CYP3A4 genetic variant and clinical presentation in African-American prostate cancer patients. Cancer Epidemiol Biomarkers Prev. 1999;8(10):901-5. PubMed PMID: 10548319.

64. Rebbeck TR, Jaffe JM, Walker AH, Wein AJ, Malkowicz SB. Modification of clinical presentation of prostate tumors by a novel genetic variant in CYP3A4. Journal of the National Cancer Institute. 1998;90(16):1225-9. PubMed PMID: 9719084.

65. Savas U, Hsu MH, Griffin KJ, Bell DR, Johnson EF. Conditional regulation of the human CYP4X1 and CYP4Z1 genes. Arch Biochem Biophys. 2005;436(2):377-85. doi: 10.1016/j.abb.2005.02.022. PubMed PMID: 15797250.

66. Lejeune FJ, Ruegg C, Lienard D. Clinical applications of TNF-alpha in cancer. Curr Opin Immunol. 1998;10(5):573-80. PubMed PMID: 9794839.

67. Balkwill F. Tumor necrosis factor or tumor promoting factor? Cytokine & growth factor reviews. 2002;13(2):135-41. PubMed PMID: 11900989.

68. Balkwill F, Mantovani A. Inflammation and cancer: back to Virchow? Lancet. 2001;357(9255):539-45. doi: 10.1016/S0140-6736(00)04046-0. PubMed PMID: 11229684.

69. Arias JI, Aller MA, Sanchez-Patan F, Arias J. Inflammation and cancer: is trophism the link? Surg Oncol. 2006;15(4):235-42. doi: 10.1016/j.suronc.2007.02.001. PubMed PMID: 17400443.

70. Rivas MA, Carnevale RP, Proietti CJ, Rosemblit C, Beguelin W, Salatino M, et al. TNF alpha acting on TNFR1 promotes breast cancer growth via p42/P44 MAPK, JNK, Akt and NF-kappa B-dependent pathways. Exp Cell Res. 2008;314(3):509-29. doi: 10.1016/j.yexcr.2007.10.005. PubMed PMID: 18061162.

71. Xu F, Zhou G, Han S, Yuan W, Chen S, Fu Z, et al. Association of TNF-alpha, TNFRSF1A and TNFRSF1B gene polymorphisms with the risk of sporadic breast cancer in northeast Chinese Han women. PLoS One. 2014;9(7):e101138. doi: 10.1371/journal.pone.0101138. PubMed PMID: 25010932; PubMed Central PMCID: PMC4091942.

72. Vanhaesebroeck B, Guillermet-Guibert J, Graupera M, Bilanges B. The emerging mechanisms of isoform-specific PI3K signalling. Nat Rev Mol Cell Biol. 2010;11(5):329-41. doi: 10.1038/nrm2882. PubMed PMID: 20379207.

73. Hsu J, Shi Y, Krajewski S, Renner S, Fisher M, Reed JC, et al. The AKT kinase is activated in multiple myeloma tumor cells. Blood. 2001;98(9):2853-5. PubMed PMID: 11675360.

74. Zollinger A, Stuhmer T, Chatterjee M, Gattenlohner S, Haralambieva E, Muller-Hermelink HK, et al. Combined functional and molecular analysis of tumor cell signaling defines 2 distinct myeloma subgroups: Akt-dependent and Akt-independent multiple myeloma. Blood. 2008;112(8):3403-11. doi: 10.1182/blood-2007-11-119362. PubMed PMID: 18635812.

75. Baumann P, Mandl-Weber S, Oduncu F, Schmidmaier R. The novel orally bioavailable inhibitor of phosphoinositol-3-kinase and mammalian target of rapamycin, NVP-BEZ235, inhibits growth and proliferation in multiple myeloma. Exp Cell Res. 2009;315(3):485-97. doi: 10.1016/j.yexcr.2008.11.007. PubMed PMID: 19071109.

76. Steinbrunn T, Stuhmer T, Gattenlohner S, Rosenwald A, Mottok A, Unzicker C, et al. Mutated RAS and constitutively activated Akt delineate distinct oncogenic pathways, which independently contribute to multiple myeloma cell survival. Blood. 2011;117(6):1998-2004. doi: 10.1182/blood-2010-05-284422. PubMed PMID: 21149634.

77. Ramakrishnan V, Kimlinger T, Haug J, Painuly U, Wellik L, Halling T, et al. Anti-myeloma activity of Akt inhibition is linked to the activation status of PI3K/Akt and MEK/ERK pathway. PLoS One. 2012;7(11):e50005. doi: 10.1371/journal.pone.0050005. PubMed PMID: 23185517; PubMed Central PMCID: PMC3503708.

78. Munugalavadla V, Mariathasan S, Slaga D, Du C, Berry L, Del Rosario G, et al. The PI3K inhibitor GDC-0941 combines with existing clinical regimens for superior activity in multiple myeloma. Oncogene. 2014;33(3):316-25. doi: 10.1038/onc.2012.594. PubMed PMID: 23318440.

79. Vivanco I, Sawyers CL. The phosphatidylinositol 3-Kinase AKT pathway in human cancer. Nat Rev Cancer. 2002;2(7):489-501. doi: 10.1038/nrc839. PubMed PMID: 12094235.

80. Wetzker R, Rommel C. Phosphoinositide 3-kinases as targets for therapeutic intervention. Curr Pharm Des. 2004;10(16):1915-22. PubMed PMID: 15180528.

81. Bartholomeusz C, Gonzalez-Angulo AM. Targeting the PI3K signaling pathway in cancer therapy. Expert opinion on therapeutic targets. 2012;16(1):121-30. doi: 10.1517/14728222.2011.644788. PubMed PMID: 22239433.

82. Crowder RJ, Phommaly C, Tao Y, Hoog J, Luo J, Perou CM, et al. PIK3CA and PIK3CB inhibition produce synthetic lethality when combined with estrogen deprivation in estrogen receptor-positive breast cancer. Cancer Res. 2009;69(9):3955-62. doi: 10.1158/0008-5472.CAN-08-4450. PubMed PMID: 19366795; PubMed Central PMCID: PMC2811393.

83. Toker A, Yoeli-Lerner M. Akt signaling and cancer: surviving but not moving on. Cancer Res. 2006;66(8):3963-6. doi: 10.1158/0008-5472.CAN-06-0743. PubMed PMID: 16618711.

84. Li H, Zeng J, Shen K. PI3K/AKT/mTOR signaling pathway as a therapeutic target for ovarian cancer. Archives of gynecology and obstetrics. 2014;290(6):1067-78. doi: 10.1007/s00404-014-3377-3. PubMed PMID: 25086744.

85. Angulo I, Vadas O, Garcon F, Banham-Hall E, Plagnol V, Leahy TR, et al. Phosphoinositide 3-kinase delta gene mutation predisposes to respiratory infection and airway damage. Science. 2013;342(6160):866-71. doi: 10.1126/science.1243292. PubMed PMID: 24136356; PubMed Central PMCID: PMC3930011.

86. Lucas CL, Kuehn HS, Zhao F, Niemela JE, Deenick EK, Palendira U, et al. Dominant-activating germline mutations in the gene encoding the PI(3)K catalytic subunit p110delta result in T cell senescence and human immunodeficiency. Nat Immunol. 2014;15(1):88-97. doi: 10.1038/ni.2771. PubMed PMID: 24165795; PubMed Central PMCID: PMC4209962.

87. Edling CE, Selvaggi F, Buus R, Maffucci T, Di Sebastiano P, Friess H, et al. Key role of phosphoinositide 3-kinase class IB in pancreatic cancer. Clin Cancer Res. 2010;16(20):4928-37. doi: 10.1158/1078-0432.CCR-10-1210. PubMed PMID: 20876794.

88. Brazzatti JA, Klingler-Hoffmann M, Haylock-Jacobs S, Harata-Lee Y, Niu M, Higgins MD, et al. Differential roles for the p101 and p84 regulatory subunits of PI3Kgamma in tumor growth and metastasis. Oncogene. 2012;31(18):2350-61. doi: 10.1038/onc.2011.414. PubMed PMID: 21996737.

89. Zhang S, Chung WC, Wu G, Egan SE, Miele L, Xu K. Manic fringe promotes a claudin-low breast cancer phenotype through notch-mediated PIK3CG induction. Cancer Res. 2015;75(10):1936-43. doi: 10.1158/0008-5472.CAN-14-3303. PubMed PMID: 25808869; PubMed Central PMCID: PMC4433600.

90. Haataja L, Groffen J, Heisterkamp N. Characterization of RAC3, a novel member of the Rho family. J Biol Chem. 1997;272(33):20384-8. PubMed PMID: 9252344.

91. Van Aelst L, D'Souza-Schorey C. Rho GTPases and signaling networks. Genes & development. 1997;11(18):2295-322. PubMed PMID: 9308960.

92. Mira JP, Benard V, Groffen J, Sanders LC, Knaus UG. Endogenous, hyperactive Rac3 controls proliferation of breast cancer cells by a p21-activated kinase-dependent pathway. Proc Natl Acad Sci U S A. 2000;97(1):185-9. PubMed PMID: 10618392; PubMed Central PMCID: PMC26637.

93. Wang S, Ghosh RN, Chellappan SP. Raf-1 physically interacts with Rb and regulates its function: a link between mitogenic signaling and cell cycle regulation. Mol Cell Biol. 1998;18(12):7487-98. PubMed PMID: 9819434; PubMed Central PMCID: PMC109329.

94. Tjandra H, Compton J, Kellogg D. Control of mitotic events by the Cdc42 GTPase, the Clb2 cyclin and a member of the PAK kinase family. Curr Biol. 1998;8(18):991-1000. PubMed PMID: 9740799.

95. Hwang SL, Chang JH, Cheng TS, Sy WD, Lieu AS, Lin CL, et al. Expression of Rac3 in human brain tumors. Journal of clinical neuroscience : official journal of the Neurosurgical Society of Australasia. 2005;12(5):571-4. doi: 10.1016/j.jocn.2004.08.013. PubMed PMID: 15993075.

96. Baugher PJ, Krishnamoorthy L, Price JE, Dharmawardhane SF. Rac1 and Rac3 isoform activation is involved in the invasive and metastatic phenotype of human breast cancer cells. Breast cancer research : BCR. 2005;7(6):R965-74. doi: 10.1186/bcr1329. PubMed PMID: 16280046; PubMed Central PMCID: PMC1410764.

97. Liu TQ, Wang GB, Li ZJ, Tong XD, Liu HX. Silencing of Rac3 inhibits proliferation and induces apoptosis of human lung cancer cells. Asian Pac J Cancer Prev. 2015;16(7):3061-5. PubMed PMID: 25854406.

98. Gauchotte G, Vigouroux C, Rech F, Battaglia-Hsu SF, Soudant M, Pinelli C, et al. Expression of minichromosome maintenance MCM6 protein in meningiomas is strongly correlated with histologic grade and clinical outcome. The American journal of surgical pathology. 2012;36(2):283-91. doi: 10.1097/PAS.0b013e318235ee03. PubMed PMID: 22020044.

99. Kikuchi J, Kinoshita I, Shimizu Y, Kikuchi E, Takeda K, Aburatani H, et al. Minichromosome maintenance (MCM) protein 4 as a marker for proliferation and its clinical and clinicopathological significance in non-small cell lung cancer. Lung Cancer. 2011;72(2):229-37. doi: 10.1016/j.lungcan.2010.08.020. PubMed PMID: 20884074.

100. Fujioka S, Shomori K, Nishihara K, Yamaga K, Nosaka K, Araki K, et al. Expression of minichromosome maintenance 7 (MCM7) in small lung adenocarcinomas (pT1): Prognostic implication. Lung Cancer. 2009;65(2):223-9. doi: 10.1016/j.lungcan.2008.11.007. PubMed PMID: 19144445.

101. Werynska B, Pula B, Muszczynska-Bernhard B, Piotrowska A, Jethon A, Podhorska-Okolow M, et al. Correlation between expression of metallothionein and expression of Ki-67 and MCM-2 proliferation markers in non-small cell lung cancer. Anticancer Res. 2011;31(9):2833-9. PubMed PMID: 21868526.

102. Vigouroux C, Casse JM, Battaglia-Hsu SF, Brochin L, Luc A, Paris C, et al. Methyl(R217)HuR and MCM6 are inversely correlated and are prognostic markers in non small cell lung carcinoma. Lung Cancer. 2015;89(2):189-96. doi: 10.1016/j.lungcan.2015.05.008. PubMed PMID: 26013954.

103. Lieberman JR, Daluiski A, Einhorn TA. The role of growth factors in the repair of bone. Biology and clinical applications. The Journal of bone and joint surgery American volume. 2002;84-A(6):1032-44. PubMed PMID: 12063342.

104. Brunet LJ, McMahon JA, McMahon AP, Harland RM. Noggin, cartilage morphogenesis, and joint formation in the mammalian skeleton. Science. 1998;280(5368):1455-7. PubMed PMID: 9603738.

105. Langenfeld EM, Calvano SE, Abou-Nukta F, Lowry SF, Amenta P, Langenfeld J. The mature bone morphogenetic protein-2 is aberrantly expressed in non-small cell lung carcinomas and stimulates tumor growth of A549 cells. Carcinogenesis. 2003;24(9):1445-54. doi: 10.1093/carcin/bgg100. PubMed PMID: 12819188.

106. Tarragona M, Pavlovic M, Arnal-Estape A, Urosevic J, Morales M, Guiu M, et al. Identification of NOG as a specific breast cancer bone metastasis-supporting gene. J Biol Chem. 2012;287(25):21346-55. doi: 10.1074/jbc.M112.355834. PubMed PMID: 22547073; PubMed Central PMCID: PMC3375555.

107. Haudenschild DR, Palmer SM, Moseley TA, You Z, Reddi AH. Bone morphogenetic protein (BMP)-6 signaling and BMP antagonist noggin in prostate cancer. Cancer Res. 2004;64(22):8276-84. doi: 10.1158/0008-5472.CAN-04-2251. PubMed PMID: 15548695.

108. Sarvaiya PJ, Guo D, Ulasov I, Gabikian P, Lesniak MS. Chemokines in tumor progression and metastasis. Oncotarget. 2013;4(12):2171-85. doi: 10.18632/oncotarget.1426. PubMed PMID: 24259307; PubMed Central PMCID: PMC3926818.

109. Raman D, Sobolik-Delmaire T, Richmond A. Chemokines in health and disease. Exp Cell Res. 2011;317(5):575-89. doi: 10.1016/j.yexcr.2011.01.005. PubMed PMID: 21223965; PubMed Central PMCID: PMC3063402.

110. Walser TC, Ma X, Kundu N, Dorsey R, Goloubeva O, Fulton AM. Immune-mediated modulation of breast cancer growth and metastasis by the chemokine Mig (CXCL9) in a murine model. Journal of immunotherapy. 2007;30(5):490-8. doi: 10.1097/CJI.0b013e318031b551. PubMed PMID: 17589289.

111. Walser TC, Rifat S, Ma X, Kundu N, Ward C, Goloubeva O, et al. Antagonism of CXCR3 inhibits lung metastasis in a murine model of metastatic breast cancer. Cancer Res. 2006;66(15):7701-7. doi: 10.1158/0008-5472.CAN-06-0709. PubMed PMID: 16885372.

112. Kawada K, Hosogi H, Sonoshita M, Sakashita H, Manabe T, Shimahara Y, et al. Chemokine receptor CXCR3 promotes colon cancer metastasis to lymph nodes. Oncogene. 2007;26(32):4679-88. doi: DOI 10.1038/sj.onc.1210267. PubMed PMID: WOS:000248037900008.

113. Ma X, Norsworthy K, Kundu N, Rodgers WH, Gimotty PA, Goloubeva O, et al. CXCR3 expression is associated with poor survival in breast cancer and promotes metastasis in a murine model. Mol Cancer Ther. 2009;8(3):490-8. doi: 10.1158/1535-7163.MCT-08-0485. PubMed PMID: 19276169.

114. Cambien B, Karimdjee BF, Richard-Fiardo P, Bziouech H, Barthel R, Millet MA, et al. Organ-specific inhibition of metastatic colon carcinoma by CXCR3 antagonism. British journal of cancer. 2009;100(11):1755-64. doi: 10.1038/sj.bjc.6605078. PubMed PMID: 19436305; PubMed Central PMCID: PMC2695685.

115. Pradelli E, Karimdjee-Soilihi B, Michiels JF, Ricci JE, Millet MA, Vandenbos F, et al. Antagonism of chemokine receptor CXCR3 inhibits osteosarcoma metastasis to lungs. International journal of cancer Journal international du cancer. 2009;125(11):2586-94. doi: 10.1002/ijc.24665. PubMed PMID: 19544560; PubMed Central PMCID: PMC2772145.

116. Zhu G, Yan HH, Pang Y, Jian J, Achyut BR, Liang X, et al. CXCR3 as a molecular target in breast cancer metastasis: inhibition of tumor cell migration and promotion of host anti-tumor immunity. Oncotarget. 2015;6(41):43408-19. doi: 10.18632/oncotarget.6125. PubMed PMID: 26485767.
