# Supplementary material for: An integrated method for the identification of novel genes related to oral cancer
Source: PLoS One. 2017 Apr 6;12(4):e0175185. doi: 10.1371/journal.pone.0175185 (PMC5383255; doi:10.1371/journal.pone.0175185)
Supplement: S1 Table — (DOCX) [file pone.0175185.s002.docx]

**S1 Table.** 202 genes related to oral cancer and their sources

| **Gene symbol** | **Source** | |
| --- | --- | --- |
| ABCB5 | UniProt | |
| ABO | NCI | |
| ACTN4 | UniProt | |
| ADA | NCI | |
| ADH1C | NCI | |
| ANO1 | UniProt | |
| APC | NCI | |
| APP | NCI | |
| ATG3 | UniProt | |
| ATP7B | NCI | |
| AXIN1 | NCI | |
| BAG1 | NCI | |
| BAX | NCI | |
| BCL2 | NCI | |
| BMI1 | NCI | |
| BMP2 | NCI | |
| BMP5 | NCI | |
| BRCA1 | NCI | |
| BRCA2 | NCI | |
| CA9 | NCI | |
| CAPG | NCI | |
| CATA | UniProt | |
| CAV1 | NCI | |
| CCL19 | NCI | |
| CCL3 | NCI | |
| CCND1 | NCI | |
| CD1A | NCI | |
| CD24 | NCI | |
| CD34 | NCI | |
| CD40 | NCI | |
| CD44 | NCI | |
| CD46 | NCI | |
| CD55 | NCI | |
| CD59 | NCI | |
| CD68 | NCI | |
| CD80 | NCI | |
| CD86 | NCI | |
| CD9 | NCI | |
| CDK1 | NCI | |
| CDK2 | NCI | |
| CDK2AP1 | TSGene | |
| CDK4 | NCI | |
| CDK6 | NCI | |
| CDKA1 | UniProt | |
| CDKN2A | NCI | |
| CO7A1 | UniProt | |
| COX2 | NCI | |
| CRNN | UniProt | |
| CSMD1 | NCI,UniProt | |
| CTGF | NCI | |
| CTLA4 | NCI | |
| CXCL10 | NCI | |
| CXCL9 | NCI | |
| CXCR4 | NCI | |
| CXL14 | UniProt | |
| CYP1A1 | NCI | |
| CYP1B1 | NCI | |
| CYP2D6 | NCI | |
| CYP2E1 | NCI | |
| CYP3A5 | NCI | |
| CYTS | UniProt | |
| DCC | NCI | |
| DCNL1 | UniProt | |
| DDX59 | UniProt | |
| DHI1 | UniProt | |
| DMBT1 | NCI | |
| DNMT1 | NCI | |
| DNMT3A | NCI | |
| DNMT3B | NCI | |
| EGFR | NCI | |
| EGR1 | NCI | |
| EPS8 | UniProt | |
| ERBB2 | NCI | |
| ERBB3 | NCI | |
| ETS1 | NCI | |
| FAS | NCI | |
| FAT1 | TSGene | |
| FEZ1 | NCI | |
| FGF1 | NCI | |
| FGF10 | UniProt | |
| FGF3 | NCI | |
| FGFR1 | NCI | |
| FGFR3 | NCI | |
| FHIT | NCI | |
| FRA11F | NCI | |
| FRA16D | NCI | |
| FYN | NCI | |
| GSDMB | UniProt | |
| GSTM1 | NCI | |
| GSTP1 | NCI | |
| GSTT1 | NCI | |
| HAX1 | UniProt | |
| HMGA2 | NCI | |
| HNP1 | NCI | |
| HOP | UniProt | |
| ICAM1 | NCI | |
| IGF2 | NCI | |
| IGF2R | NCI | |
| IL10 | NCI | |
| IL15 | NCI | |
| IL18 | NCI | |
| IL2 | NCI | |
| IL6 | NCI | |
| IL6R | NCI | |
| IL8 | NCI | |
| ING1 | NCI | |
| ING5 | TSGene | |
| IRF8 | UniProt | |
| K1C16 | UniProt | |
| K1C19 | UniProt | |
| K1C20 | UniProt | |
| K22O | UniProt | |
| LDB1 | NCI | |
| LIMA1 | UniProt | |
| LIN7C | NCI | |
| LMO4 | NCI | |
| LRP12 | NCI | |
| M3K9 | UniProt | |
| MCL1 | NCI | |
| MCM2 | NCI | |
| MDM2 | NCI | |
| MGMT | NCI | |
| MICA | NCI | |
| MK06 | UniProt | |
| MMP1 | NCI | |
| MMP2 | NCI | |
| MMP3 | NCI | |
| MMP9 | NCI | |
| MTHFR | NCI | |
| MTNR1A | NCI | |
| MUC1 | NCI | |
| MUTYH | NCI | |
| NAT1 | NCI | |
| NAT2 | NCI | |
| NDRG1 | NCI | |
| NDRG2 | TSGene | |
| NFKB1 | NCI | |
| NOS2 | NCI | |
| NOTCH1 | NCI | |
| NSD1 | UniProt | |
| OGG1 | NCI | |
| ORAOV1 | NCI | |
| ORAV1 | UniProt | |
| P53 | UniProt | |
| PAR1 | NCI | |
| PCNA | NCI | |
| PDCD5 | NCI | |
| PDK2 | UniProt | |
| PDPN | UniProt | |
| PGFRL | UniProt | |
| PGP | NCI | |
| PIK3CA | NCI | |
| PIM1 | NCI | |
| PIN1 | NCI | |
| PLPL3 | UniProt | |
| PRB4 | UniProt | |
| PRL | NCI | |
| PRTFDC1 | NCI | |
| PTEN | NCI | |
| PTK6 | UniProt | |
| RAC1 | NCI | |
| RAD21 | NCI | |
| RAGE | NCI | |
| RASH | UniProt | |
| RB1 | NCI,TSGene | |
| RBMX | UniProt | |
| RHDF2 | UniProt | |
| RIN1 | NCI | |
| RPL14 | NCI | |
| S100A2 | NCI | |
| S100A4 | NCI | |
| SART1 | NCI | |
| SART3 | NCI | |
| SHANK2 | NCI | |
| SHH | NCI | |
| SKP2 | NCI | |
| SMAD2 | NCI | |
| SMAD4 | NCI | |
| SP1 | NCI | |
| SRPX2 | UniProt | |
| ST3 | NCI | |
| STAT3 | NCI | |
| TAP1 | NCI | |
| TAP2 | NCI | |
| TIAM1 | NCI | |
| TIMP1 | NCI | |
| TIMP2 | NCI | |
| TLR4 | NCI | |
| TMPS6 | UniProt | |
| TNF | NCI | |
| TRPV1 | NCI | |
| TSC2 | NCI | |
| VCAM1 | NCI | |
| VGFR3 | UniProt | |
| VP13A | UniProt | |
| WNT1 | NCI | |
| WRN | UniProt | |
| WWOX | NCI,TSGene | |
| XIAP | NCI | |
| XPA | NCI | |
| XRCC1 | NCI | |
| YWHAZ | NCI | |
| ABCB5 | |  |
| ABO | |  |
| ACTN4 | |  |
| ADA | |  |
| ADH1C | |  |
| ANO1 | |  |
| APC | |  |
| APP | |  |
| ATG3 | |  |
| ATP7B | |  |
| AXIN1 | |  |
| BAG1 | |  |
| BAX | |  |
| BCL2 | |  |
| BMI1 | |  |
| BMP2 | |  |
| BMP5 | |  |
| BRCA1 | |  |
| BRCA2 | |  |
| CA9 | |  |
| CAPG | |  |
| CATA | |  |
| CAV1 | |  |
| CCL19 | |  |
| CCL3 | |  |
| CCND1 | |  |
| CD1A | |  |
| CD24 | |  |
| CD34 | |  |
| CD40 | |  |
| CD44 | |  |
| CD46 | |  |
| CD55 | |  |
| CD59 | |  |
| CD68 | |  |
| CD80 | |  |
| CD86 | |  |
| CD9 | |  |
| CDK1 | |  |
| CDK2 | |  |
| CDK2AP1 | |  |
| CDK4 | |  |
| CDK6 | |  |
| CDKA1 | |  |
| CDKN2A | |  |
| CO7A1 | |  |
| COX2 | |  |
| CRNN | |  |
| CSMD1 | |  |
| CTGF | |  |
| CTLA4 | |  |
| CXCL10 | |  |
| CXCL9 | |  |
| CXCR4 | |  |
| CXL14 | |  |
| CYP1A1 | |  |
| CYP1B1 | |  |
| CYP2D6 | |  |
| CYP2E1 | |  |
| CYP3A5 | |  |
| CYTS | |  |
| DCC | |  |
| DCNL1 | |  |
| DDX59 | |  |
| DHI1 | |  |
| DMBT1 | |  |
| DNMT1 | |  |
| DNMT3A | |  |
| DNMT3B | |  |
| EGFR | |  |
| EGR1 | |  |
| EPS8 | |  |
| ERBB2 | |  |
| ERBB3 | |  |
| ETS1 | |  |
| FAS | |  |
| FAT1 | |  |
| FEZ1 | |  |
| FGF1 | |  |
| FGF10 | |  |
| FGF3 | |  |
| FGFR1 | |  |
| FGFR3 | |  |
| FHIT | |  |
| FRA11F | |  |
| FRA16D | |  |
| FYN | |  |
| GSDMB | |  |
| GSTM1 | |  |
| GSTP1 | |  |
| GSTT1 | |  |
| HAX1 | |  |
| HMGA2 | |  |
| HNP1 | |  |
| HOP | |  |
| ICAM1 | |  |
| IGF2 | |  |
| IGF2R | |  |
| IL10 | |  |
| IL15 | |  |
| IL18 | |  |
| IL2 | |  |
| IL6 | |  |
| IL6R | |  |
| IL8 | |  |
| ING1 | |  |
| ING5 | |  |
| IRF8 | |  |
| K1C16 | |  |
| K1C19 | |  |
| K1C20 | |  |
| K22O | |  |
| LDB1 | |  |
| LIMA1 | |  |
| LIN7C | |  |
| LMO4 | |  |
| LRP12 | |  |
| M3K9 | |  |
| MCL1 | |  |
| MCM2 | |  |
| MDM2 | |  |
| MGMT | |  |
| MICA | |  |
| MK06 | |  |
| MMP1 | |  |
| MMP2 | |  |
| MMP3 | |  |
| MMP9 | |  |
| MTHFR | |  |
| MTNR1A | |  |
| MUC1 | |  |
| MUTYH | |  |
| NAT1 | |  |
| NAT2 | |  |
| NDRG1 | |  |
| NDRG2 | |  |
| NFKB1 | |  |
| NOS2 | |  |
| NOTCH1 | |  |
| NSD1 | |  |
| OGG1 | |  |
| ORAOV1 | |  |
| ORAV1 | |  |
| P53 | |  |
| PAR1 | |  |
| PCNA | |  |
| PDCD5 | |  |
| PDK2 | |  |
| PDPN | |  |
| PGFRL | |  |
| PGP | |  |
| PIK3CA | |  |
| PIM1 | |  |
| PIN1 | |  |
| PLPL3 | |  |
| PRB4 | |  |
| PRL | |  |
| PRTFDC1 | |  |
| PTEN | |  |
| PTK6 | |  |
| RAC1 | |  |
| RAD21 | |  |
| RAGE | |  |
| RASH | |  |
| RB1 | |  |
| RBMX | |  |
| RHDF2 | |  |
| RIN1 | |  |
| RPL14 | |  |
| S100A2 | |  |
| S100A4 | |  |
| SART1 | |  |
| SART3 | |  |
| SHANK2 | |  |
| SHH | |  |
| SKP2 | |  |
| SMAD2 | |  |
| SMAD4 | |  |
| SP1 | |  |
| SRPX2 | |  |
| ST3 | |  |
| STAT3 | |  |
| TAP1 | |  |
| TAP2 | |  |
| TIAM1 | |  |
| TIMP1 | |  |
| TIMP2 | |  |
| TLR4 | |  |
| TMPS6 | |  |
| TNF | |  |
| TRPV1 | |  |
| TSC2 | |  |
| VCAM1 | |  |
| VGFR3 | |  |
| VP13A | |  |
| WNT1 | |  |
| WRN | |  |
| WWOX | |  |
| XIAP | |  |
| XPA | |  |
| XRCC1 | |  |
| YWHAZ | |  |
