# Supplementary material for: An integrated method for the identification of novel genes related to oral cancer
Source: PLoS One. 2017 Apr 6;12(4):e0175185. doi: 10.1371/journal.pone.0175185 (PMC5383255; doi:10.1371/journal.pone.0175185)
Supplement: S2 Table — (DOCX) [file pone.0175185.s003.docx]

**S2 Table.** The performance of the RWR-based method with different combinations of parameters

| **Threshold parameter of probability $p_{RWR}^{p}$** | **Threshold parameter of maximum interaction score $p_{RWR}^{MIS}$** | **Threshold parameter of maximum function score $p_{RWR}^{MFS}$** | **Recall** | **Precision** | **F1-measure-R** |
| --- | --- | --- | --- | --- | --- |
| 0.00006 | 400 | 0.8 | 0.26087 | 0.003254 | 0.001677 |
| 0.00006 | 700 | 0.8 | 0.26087 | 0.003254 | 0.001677 |
| 0.00007 | 400 | 0.8 | 0.23913 | 0.00352 | 0.001659 |
| 0.00007 | 700 | 0.8 | 0.23913 | 0.00352 | 0.001659 |
| 0.00006 | 900 | 0.8 | 0.255435 | 0.003188 | 0.001608 |
| 0.00007 | 900 | 0.8 | 0.233696 | 0.00344 | 0.001584 |
| 0.00001 | 700 | 0.8 | 0.277174 | 0.002801 | 0.001537 |
| 0.00002 | 700 | 0.8 | 0.277174 | 0.002801 | 0.001537 |
| 0.00001 | 400 | 0.8 | 0.277174 | 0.002801 | 0.001537 |
| 0.00002 | 400 | 0.8 | 0.277174 | 0.002801 | 0.001537 |
| 0.00004 | 400 | 0.8 | 0.271739 | 0.002803 | 0.001508 |
| 0.00004 | 700 | 0.8 | 0.271739 | 0.002803 | 0.001508 |
| 0.00005 | 400 | 0.8 | 0.266304 | 0.002839 | 0.001496 |
| 0.00005 | 700 | 0.8 | 0.266304 | 0.002839 | 0.001496 |
| 0.00003 | 700 | 0.8 | 0.271739 | 0.002747 | 0.001478 |
| 0.00003 | 400 | 0.8 | 0.271739 | 0.002746 | 0.001478 |
| 0.00004 | 900 | 0.8 | 0.266304 | 0.002749 | 0.001449 |
| 0.00005 | 900 | 0.8 | 0.26087 | 0.002783 | 0.001437 |
| 0.00001 | 900 | 0.8 | 0.266304 | 0.002721 | 0.001435 |
| 0.00002 | 900 | 0.8 | 0.266304 | 0.002721 | 0.001435 |
| 0.00003 | 900 | 0.8 | 0.266304 | 0.002721 | 0.001435 |
| 0.00008 | 400 | 0.8 | 0.206522 | 0.003455 | 0.001404 |
| 0.00008 | 700 | 0.8 | 0.206522 | 0.003455 | 0.001404 |
| 0.0001 | 400 | 0.8 | 0.173913 | 0.004078 | 0.001386 |
| 0.0001 | 700 | 0.8 | 0.173913 | 0.004078 | 0.001386 |
| 0.00009 | 400 | 0.8 | 0.195652 | 0.003574 | 0.001373 |
| 0.00009 | 700 | 0.8 | 0.195652 | 0.003574 | 0.001373 |
| 0.00006 | 700 | 0.7 | 0.358696 | 0.001914 | 0.001366 |
| 0.00006 | 900 | 0.7 | 0.347826 | 0.001973 | 0.001365 |
| 0.00007 | 700 | 0.7 | 0.326087 | 0.002098 | 0.001359 |
| 0.00007 | 900 | 0.7 | 0.315217 | 0.002155 | 0.001349 |
| 0.00007 | 400 | 0.7 | 0.326087 | 0.002071 | 0.001343 |
| 0.00006 | 400 | 0.7 | 0.358696 | 0.001874 | 0.001337 |
| 0.00008 | 900 | 0.8 | 0.201087 | 0.003364 | 0.001331 |
| 0.00005 | 700 | 0.7 | 0.38587 | 0.001702 | 0.001308 |
| 0.00005 | 900 | 0.7 | 0.375 | 0.001748 | 0.001305 |
| 0.0001 | 900 | 0.8 | 0.168478 | 0.003952 | 0.001301 |
| 0.00009 | 900 | 0.8 | 0.190217 | 0.003475 | 0.001298 |
| 0.00007 | 900 | 0.6 | 0.353261 | 0.001824 | 0.001282 |
| 0.00005 | 400 | 0.7 | 0.38587 | 0.001658 | 0.001274 |
| 0.00008 | 700 | 0.7 | 0.288043 | 0.002224 | 0.001271 |
| 0.00009 | 700 | 0.7 | 0.26087 | 0.00244 | 0.001261 |
| 0.00008 | 400 | 0.7 | 0.288043 | 0.002191 | 0.001253 |
| 0.00009 | 400 | 0.7 | 0.26087 | 0.00242 | 0.001251 |
| 0.00009 | 900 | 0.7 | 0.255435 | 0.002464 | 0.001247 |
| 0.00007 | 700 | 0.6 | 0.36413 | 0.001718 | 0.001245 |
| 0.00004 | 700 | 0.7 | 0.396739 | 0.001557 | 0.001231 |
| 0.00007 | 400 | 0.6 | 0.36413 | 0.001694 | 0.001228 |
| 0.00008 | 900 | 0.7 | 0.277174 | 0.002227 | 0.001225 |
| 0.00004 | 900 | 0.7 | 0.38587 | 0.001591 | 0.001223 |
| 0.00006 | 900 | 0.6 | 0.391304 | 0.001561 | 0.001217 |
| 0.00007 | 900 | 0.5 | 0.353261 | 0.001722 | 0.001211 |
| 0.00004 | 400 | 0.7 | 0.396739 | 0.001521 | 0.001203 |
| 0.00006 | 700 | 0.6 | 0.407609 | 0.00148 | 0.001202 |
| 0.00007 | 700 | 0.5 | 0.369565 | 0.001626 | 0.001196 |
| 0.00007 | 900 | 0.4 | 0.353261 | 0.001689 | 0.001187 |
| 0.00003 | 900 | 0.7 | 0.38587 | 0.001525 | 0.001173 |
| 0.00006 | 400 | 0.6 | 0.407609 | 0.001443 | 0.001172 |
| 0.00007 | 400 | 0.5 | 0.369565 | 0.00159 | 0.00117 |
| 0.00007 | 700 | 0.4 | 0.369565 | 0.001584 | 0.001166 |
| 0.00007 | 900 | 0 | 0.353261 | 0.001657 | 0.001165 |
| 0.00007 | 900 | 0.1 | 0.353261 | 0.001657 | 0.001165 |
| 0.00007 | 900 | 0.2 | 0.353261 | 0.001657 | 0.001165 |
| 0.00007 | 900 | 0.3 | 0.353261 | 0.001657 | 0.001165 |
| 0.00006 | 700 | 0.5 | 0.418478 | 0.001394 | 0.001163 |
| 0.00001 | 700 | 0.7 | 0.402174 | 0.001449 | 0.001161 |
| 0.00002 | 700 | 0.7 | 0.402174 | 0.001449 | 0.001161 |
| 0.00001 | 900 | 0.7 | 0.38587 | 0.001505 | 0.001157 |
| 0.00002 | 900 | 0.7 | 0.38587 | 0.001505 | 0.001157 |
| 0.00003 | 700 | 0.7 | 0.396739 | 0.001463 | 0.001156 |
| 0.00006 | 900 | 0.5 | 0.391304 | 0.001462 | 0.00114 |
| 0.00002 | 400 | 0.7 | 0.402174 | 0.001418 | 0.001137 |
| 0.00007 | 400 | 0.4 | 0.369565 | 0.001544 | 0.001136 |
| 0.00001 | 400 | 0.7 | 0.402174 | 0.001413 | 0.001133 |
| 0.00003 | 400 | 0.7 | 0.396739 | 0.001431 | 0.001131 |
| 0.00009 | 900 | 0.6 | 0.271739 | 0.002096 | 0.00113 |
| 0.00006 | 400 | 0.5 | 0.418478 | 0.001351 | 0.001127 |
| 0.00006 | 700 | 0.4 | 0.418478 | 0.00135 | 0.001127 |
| 0.00009 | 700 | 0.6 | 0.277174 | 0.002039 | 0.001122 |
| 0.00006 | 900 | 0.4 | 0.391304 | 0.001436 | 0.00112 |
| 0.00007 | 700 | 0.2 | 0.369565 | 0.001519 | 0.001118 |
| 0.00007 | 700 | 0.3 | 0.369565 | 0.001519 | 0.001118 |
| 0.0001 | 400 | 0.7 | 0.217391 | 0.002602 | 0.001118 |
| 0.0001 | 700 | 0.7 | 0.217391 | 0.002602 | 0.001118 |
| 0.00007 | 700 | 0 | 0.369565 | 0.001513 | 0.001113 |
| 0.00007 | 700 | 0.1 | 0.369565 | 0.001513 | 0.001113 |
| 0.00008 | 700 | 0.6 | 0.304348 | 0.001838 | 0.001112 |
| 0.00009 | 400 | 0.6 | 0.277174 | 0.002011 | 0.001107 |
| 0.00008 | 700 | 0.5 | 0.309783 | 0.001795 | 0.001106 |
| 0.00009 | 900 | 0.5 | 0.271739 | 0.002048 | 0.001105 |
| 0.00006 | 900 | 0.2 | 0.391304 | 0.001415 | 0.001103 |
| 0.00006 | 900 | 0.3 | 0.391304 | 0.001415 | 0.001103 |
| 0.0001 | 900 | 0.7 | 0.211957 | 0.002629 | 0.001101 |
| 0.00005 | 900 | 0.6 | 0.418478 | 0.001319 | 0.001101 |
| 0.00006 | 900 | 0 | 0.391304 | 0.00141 | 0.0011 |
| 0.00006 | 900 | 0.1 | 0.391304 | 0.00141 | 0.0011 |
| 0.00008 | 900 | 0.6 | 0.293478 | 0.00188 | 0.001097 |
| 0.00008 | 400 | 0.6 | 0.304348 | 0.001806 | 0.001093 |
| 0.00009 | 700 | 0.5 | 0.277174 | 0.001981 | 0.00109 |
| 0.00006 | 400 | 0.4 | 0.418478 | 0.001306 | 0.001089 |
| 0.00006 | 700 | 0.2 | 0.418478 | 0.0013 | 0.001085 |
| 0.00006 | 700 | 0.3 | 0.418478 | 0.0013 | 0.001085 |
| 0.00008 | 400 | 0.5 | 0.309783 | 0.001755 | 0.001081 |
| 0.00006 | 700 | 0 | 0.418478 | 0.001292 | 0.001078 |
| 0.00006 | 700 | 0.1 | 0.418478 | 0.001292 | 0.001078 |
| 0.00009 | 900 | 0.4 | 0.271739 | 0.001988 | 0.001073 |
| 0.00005 | 700 | 0.6 | 0.434783 | 0.001235 | 0.00107 |
| 0.00008 | 700 | 0.4 | 0.309783 | 0.001735 | 0.001069 |
| 0.00009 | 400 | 0.5 | 0.277174 | 0.001941 | 0.001068 |
| 0.00007 | 400 | 0.2 | 0.369565 | 0.001447 | 0.001065 |
| 0.00007 | 400 | 0.3 | 0.369565 | 0.001447 | 0.001065 |
| 0.00008 | 900 | 0.5 | 0.293478 | 0.001822 | 0.001063 |
| 0.00007 | 400 | 0 | 0.369565 | 0.001436 | 0.001057 |
| 0.00007 | 400 | 0.1 | 0.369565 | 0.001436 | 0.001057 |
| 0.00009 | 700 | 0.4 | 0.277174 | 0.001912 | 0.001053 |
| 0.00009 | 900 | 0 | 0.271739 | 0.001932 | 0.001043 |
| 0.00009 | 900 | 0.1 | 0.271739 | 0.001932 | 0.001043 |
| 0.00009 | 900 | 0.2 | 0.271739 | 0.001932 | 0.001043 |
| 0.00009 | 900 | 0.3 | 0.271739 | 0.001932 | 0.001043 |
| 0.00008 | 400 | 0.4 | 0.309783 | 0.001688 | 0.00104 |
| 0.00008 | 900 | 0.4 | 0.293478 | 0.001778 | 0.001037 |
| 0.00005 | 400 | 0.6 | 0.434783 | 0.001191 | 0.001033 |
| 0.00006 | 400 | 0.2 | 0.418478 | 0.001236 | 0.001032 |
| 0.00006 | 400 | 0.3 | 0.418478 | 0.001236 | 0.001032 |
| 0.00009 | 400 | 0.4 | 0.277174 | 0.001862 | 0.001026 |
| 0.00006 | 400 | 0 | 0.418478 | 0.001226 | 0.001023 |
| 0.00006 | 400 | 0.1 | 0.418478 | 0.001226 | 0.001023 |
| 0.00004 | 900 | 0.6 | 0.434783 | 0.001175 | 0.001019 |
| 0.00005 | 900 | 0.5 | 0.423913 | 0.001201 | 0.001016 |
| 0.00008 | 700 | 0.2 | 0.309783 | 0.001644 | 0.001013 |
| 0.00008 | 700 | 0.3 | 0.309783 | 0.001644 | 0.001013 |
| 0.00008 | 900 | 0 | 0.293478 | 0.001736 | 0.001013 |
| 0.00008 | 900 | 0.1 | 0.293478 | 0.001736 | 0.001013 |
| 0.00008 | 900 | 0.2 | 0.293478 | 0.001736 | 0.001013 |
| 0.00008 | 900 | 0.3 | 0.293478 | 0.001736 | 0.001013 |
| 0.00004 | 700 | 0.6 | 0.456522 | 0.001109 | 0.00101 |
| 0.00008 | 700 | 0 | 0.309783 | 0.001636 | 0.001008 |
| 0.00008 | 700 | 0.1 | 0.309783 | 0.001636 | 0.001008 |
| 0.00005 | 700 | 0.5 | 0.451087 | 0.001112 | 0.001001 |
| 0.00005 | 400 | 0.4 | 0.467391 | 0.001061 | 0.000989 |
| 0.00005 | 900 | 0.4 | 0.423913 | 0.00117 | 0.000989 |
| 0.00009 | 700 | 0.2 | 0.277174 | 0.001793 | 0.000987 |
| 0.00009 | 700 | 0.3 | 0.277174 | 0.001793 | 0.000987 |
| 0.0001 | 900 | 0.6 | 0.222826 | 0.002236 | 0.000987 |
| 0.0001 | 400 | 0.6 | 0.228261 | 0.002182 | 0.000987 |
| 0.0001 | 700 | 0.6 | 0.228261 | 0.002182 | 0.000987 |
| 0.00005 | 700 | 0.4 | 0.456522 | 0.001082 | 0.000986 |
| 0.00009 | 700 | 0 | 0.277174 | 0.001781 | 0.000981 |
| 0.00009 | 700 | 0.1 | 0.277174 | 0.001781 | 0.000981 |
| 0.00004 | 400 | 0.6 | 0.456522 | 0.001069 | 0.000974 |
| 0.00005 | 900 | 0.3 | 0.423913 | 0.001151 | 0.000973 |
| 0.0001 | 700 | 0.5 | 0.228261 | 0.002141 | 0.000968 |
| 0.00005 | 900 | 0.2 | 0.423913 | 0.001145 | 0.000968 |
| 0.0001 | 900 | 0.5 | 0.222826 | 0.002192 | 0.000967 |
| 0.00005 | 900 | 0 | 0.423913 | 0.001142 | 0.000966 |
| 0.00005 | 900 | 0.1 | 0.423913 | 0.001142 | 0.000966 |
| 0.00005 | 400 | 0.5 | 0.451087 | 0.001068 | 0.000961 |
| 0.0001 | 400 | 0.5 | 0.228261 | 0.002122 | 0.00096 |
| 0.00008 | 400 | 0.2 | 0.309783 | 0.001553 | 0.000958 |
| 0.00008 | 400 | 0.3 | 0.309783 | 0.001553 | 0.000958 |
| 0.00005 | 700 | 0.3 | 0.456522 | 0.001045 | 0.000952 |
| 0.00001 | 400 | 0.9 | 0.081522 | 0.006271 | 0.000949 |
| 0.00001 | 700 | 0.9 | 0.081522 | 0.006271 | 0.000949 |
| 0.00002 | 400 | 0.9 | 0.081522 | 0.006271 | 0.000949 |
| 0.00002 | 700 | 0.9 | 0.081522 | 0.006271 | 0.000949 |
| 0.00008 | 400 | 0 | 0.309783 | 0.001538 | 0.000948 |
| 0.00008 | 400 | 0.1 | 0.309783 | 0.001538 | 0.000948 |
| 0.00005 | 700 | 0.2 | 0.456522 | 0.00104 | 0.000948 |
| 0.00003 | 900 | 0.6 | 0.434783 | 0.00109 | 0.000945 |
| 0.00005 | 400 | 0.3 | 0.467391 | 0.001013 | 0.000945 |
| 0.00005 | 700 | 0 | 0.456522 | 0.001036 | 0.000944 |
| 0.00005 | 700 | 0.1 | 0.456522 | 0.001036 | 0.000944 |
| 0.0001 | 900 | 0.4 | 0.222826 | 0.002129 | 0.00094 |
| 0.00005 | 400 | 0.2 | 0.467391 | 0.001004 | 0.000937 |
| 0.0001 | 700 | 0.4 | 0.228261 | 0.002064 | 0.000934 |
| 0.00002 | 900 | 0.6 | 0.440217 | 0.001061 | 0.000932 |
| 0.00005 | 400 | 0 | 0.467391 | 0.000998 | 0.000931 |
| 0.00005 | 400 | 0.1 | 0.467391 | 0.000998 | 0.000931 |
| 0.00009 | 400 | 0.2 | 0.277174 | 0.001684 | 0.000928 |
| 0.00009 | 400 | 0.3 | 0.277174 | 0.001684 | 0.000928 |
| 0.00001 | 900 | 0.6 | 0.440217 | 0.001056 | 0.000928 |
| 0.00004 | 900 | 0.5 | 0.445652 | 0.001034 | 0.000919 |
| 0.0001 | 400 | 0.4 | 0.228261 | 0.002027 | 0.000917 |
| 0.00009 | 400 | 0 | 0.277174 | 0.001664 | 0.000917 |
| 0.00009 | 400 | 0.1 | 0.277174 | 0.001664 | 0.000917 |
| 0.00004 | 700 | 0.4 | 0.494565 | 0.000919 | 0.000908 |
| 0.0001 | 900 | 0 | 0.222826 | 0.002052 | 0.000906 |
| 0.0001 | 900 | 0.1 | 0.222826 | 0.002052 | 0.000906 |
| 0.0001 | 900 | 0.2 | 0.222826 | 0.002052 | 0.000906 |
| 0.0001 | 900 | 0.3 | 0.222826 | 0.002052 | 0.000906 |
| 0.00003 | 700 | 0.6 | 0.456522 | 0.000988 | 0.0009 |
| 0.00004 | 700 | 0.5 | 0.478261 | 0.000942 | 0.0009 |
| 0.00004 | 400 | 0.4 | 0.505435 | 0.000889 | 0.000897 |
| 0.00002 | 700 | 0.6 | 0.467391 | 0.000955 | 0.000891 |
| 0.00001 | 700 | 0.6 | 0.467391 | 0.000949 | 0.000885 |
| 0.00004 | 900 | 0.4 | 0.445652 | 0.00099 | 0.00088 |
| 0.00004 | 700 | 0.3 | 0.494565 | 0.000887 | 0.000875 |
| 0.0001 | 700 | 0 | 0.228261 | 0.001926 | 0.000872 |
| 0.0001 | 700 | 0.1 | 0.228261 | 0.001926 | 0.000872 |
| 0.0001 | 700 | 0.2 | 0.228261 | 0.001926 | 0.000872 |
| 0.0001 | 700 | 0.3 | 0.228261 | 0.001926 | 0.000872 |
| 0.00004 | 700 | 0.2 | 0.494565 | 0.00088 | 0.000869 |
| 0.00004 | 700 | 0 | 0.494565 | 0.000876 | 0.000864 |
| 0.00004 | 700 | 0.1 | 0.494565 | 0.000876 | 0.000864 |
| 0.00004 | 400 | 0.5 | 0.478261 | 0.000903 | 0.000862 |
| 0.00004 | 900 | 0.3 | 0.445652 | 0.000968 | 0.000861 |
| 0.00004 | 400 | 0 | 0.51087 | 0.000839 | 0.000856 |
| 0.00004 | 400 | 0.1 | 0.51087 | 0.000839 | 0.000856 |
| 0.00004 | 400 | 0.3 | 0.505435 | 0.000847 | 0.000855 |
| 0.00004 | 900 | 0.2 | 0.445652 | 0.00096 | 0.000854 |
| 0.00003 | 400 | 0.6 | 0.456522 | 0.000935 | 0.000852 |
| 0.00004 | 900 | 0 | 0.445652 | 0.000958 | 0.000852 |
| 0.00004 | 900 | 0.1 | 0.445652 | 0.000958 | 0.000852 |
| 0.00004 | 400 | 0.2 | 0.505435 | 0.000836 | 0.000844 |
| 0.00002 | 400 | 0.6 | 0.467391 | 0.000896 | 0.000836 |
| 0.0001 | 400 | 0.2 | 0.228261 | 0.001833 | 0.00083 |
| 0.0001 | 400 | 0.3 | 0.228261 | 0.001833 | 0.00083 |
| 0.00001 | 900 | 0.9 | 0.076087 | 0.005853 | 0.000827 |
| 0.00002 | 900 | 0.9 | 0.076087 | 0.005853 | 0.000827 |
| 0.00003 | 400 | 0.9 | 0.076087 | 0.005853 | 0.000827 |
| 0.00003 | 700 | 0.9 | 0.076087 | 0.005853 | 0.000827 |
| 0.00003 | 900 | 0.9 | 0.076087 | 0.005853 | 0.000827 |
| 0.00004 | 400 | 0.9 | 0.076087 | 0.005853 | 0.000827 |
| 0.00004 | 700 | 0.9 | 0.076087 | 0.005853 | 0.000827 |
| 0.00004 | 900 | 0.9 | 0.076087 | 0.005853 | 0.000827 |
| 0.00005 | 400 | 0.9 | 0.076087 | 0.005853 | 0.000827 |
| 0.00005 | 700 | 0.9 | 0.076087 | 0.005853 | 0.000827 |
| 0.00005 | 900 | 0.9 | 0.076087 | 0.005853 | 0.000827 |
| 0.00006 | 400 | 0.9 | 0.076087 | 0.005853 | 0.000827 |
| 0.00006 | 700 | 0.9 | 0.076087 | 0.005853 | 0.000827 |
| 0.00006 | 900 | 0.9 | 0.076087 | 0.005853 | 0.000827 |
| 0.00001 | 400 | 0.6 | 0.467391 | 0.000884 | 0.000825 |
| 0.0001 | 400 | 0 | 0.228261 | 0.001818 | 0.000824 |
| 0.0001 | 400 | 0.1 | 0.228261 | 0.001818 | 0.000824 |
| 0.00003 | 900 | 0.5 | 0.445652 | 0.000908 | 0.000808 |
| 0.00003 | 700 | 0.4 | 0.505435 | 0.000781 | 0.000788 |
| 0.00002 | 900 | 0.5 | 0.451087 | 0.000873 | 0.000786 |
| 0.00003 | 700 | 0.5 | 0.483696 | 0.000812 | 0.000784 |
| 0.00001 | 900 | 0.5 | 0.451087 | 0.000866 | 0.00078 |
| 0.00009 | 400 | 0.9 | 0.065217 | 0.006522 | 0.000773 |
| 0.00009 | 700 | 0.9 | 0.065217 | 0.006522 | 0.000773 |
| 0.00009 | 900 | 0.9 | 0.065217 | 0.006522 | 0.000773 |
| 0.00002 | 700 | 0.5 | 0.494565 | 0.000776 | 0.000767 |
| 0.00002 | 700 | 0.4 | 0.516304 | 0.000739 | 0.000762 |
| 0.00003 | 700 | 0.3 | 0.505435 | 0.000751 | 0.000758 |
| 0.00001 | 700 | 0.5 | 0.494565 | 0.000766 | 0.000756 |
| 0.00003 | 900 | 0.4 | 0.445652 | 0.000848 | 0.000754 |
| 0.00003 | 400 | 0.4 | 0.516304 | 0.000731 | 0.000754 |
| 0.00003 | 700 | 0.2 | 0.505435 | 0.000741 | 0.000748 |
| 0.00003 | 700 | 0 | 0.505435 | 0.000737 | 0.000744 |
| 0.00003 | 700 | 0.1 | 0.505435 | 0.000737 | 0.000744 |
| 0.00001 | 700 | 0.4 | 0.516304 | 0.000718 | 0.00074 |
| 0.0001 | 400 | 0.9 | 0.054348 | 0.007764 | 0.000738 |
| 0.0001 | 700 | 0.9 | 0.054348 | 0.007764 | 0.000738 |
| 0.0001 | 900 | 0.9 | 0.054348 | 0.007764 | 0.000738 |
| 0.00003 | 900 | 0.3 | 0.445652 | 0.000827 | 0.000736 |
| 0.00003 | 400 | 0.5 | 0.483696 | 0.000761 | 0.000735 |
| 0.00003 | 900 | 0.2 | 0.445652 | 0.00082 | 0.00073 |
| 0.00002 | 900 | 0.4 | 0.451087 | 0.00081 | 0.000729 |
| 0.00003 | 900 | 0 | 0.445652 | 0.000818 | 0.000728 |
| 0.00003 | 900 | 0.1 | 0.445652 | 0.000818 | 0.000728 |
| 0.00002 | 700 | 0.3 | 0.516304 | 0.000705 | 0.000727 |
| 0.00003 | 400 | 0 | 0.527174 | 0.000685 | 0.000721 |
| 0.00003 | 400 | 0.1 | 0.527174 | 0.000685 | 0.000721 |
| 0.00001 | 900 | 0.4 | 0.451087 | 0.0008 | 0.00072 |
| 0.00002 | 400 | 0.4 | 0.527174 | 0.00068 | 0.000716 |
| 0.00002 | 700 | 0.2 | 0.516304 | 0.000692 | 0.000714 |
| 0.00003 | 400 | 0.3 | 0.516304 | 0.000692 | 0.000714 |
| 0.00002 | 900 | 0.3 | 0.451087 | 0.000789 | 0.00071 |
| 0.00008 | 400 | 0.9 | 0.065217 | 0.005929 | 0.000709 |
| 0.00008 | 700 | 0.9 | 0.065217 | 0.005929 | 0.000709 |
| 0.00008 | 900 | 0.9 | 0.065217 | 0.005929 | 0.000709 |
| 0.00002 | 400 | 0.5 | 0.494565 | 0.000717 | 0.000709 |
| 0.00002 | 700 | 0 | 0.516304 | 0.000686 | 0.000708 |
| 0.00002 | 700 | 0.1 | 0.516304 | 0.000686 | 0.000708 |
| 0.00001 | 700 | 0.3 | 0.516304 | 0.000683 | 0.000705 |
| 0.00002 | 900 | 0.2 | 0.451087 | 0.000778 | 0.000701 |
| 0.00001 | 900 | 0.3 | 0.451087 | 0.000777 | 0.0007 |
| 0.00002 | 900 | 0 | 0.451087 | 0.000775 | 0.000698 |
| 0.00002 | 900 | 0.1 | 0.451087 | 0.000775 | 0.000698 |
| 0.00003 | 400 | 0.2 | 0.516304 | 0.000677 | 0.000698 |
| 0.00001 | 400 | 0.5 | 0.494565 | 0.000701 | 0.000692 |
| 0.00001 | 400 | 0.4 | 0.527174 | 0.000657 | 0.000692 |
| 0.00001 | 700 | 0.2 | 0.516304 | 0.000669 | 0.00069 |
| 0.00001 | 900 | 0.2 | 0.451087 | 0.000765 | 0.000689 |
| 0.00001 | 900 | 0 | 0.451087 | 0.000761 | 0.000686 |
| 0.00001 | 900 | 0.1 | 0.451087 | 0.000761 | 0.000686 |
| 0.00001 | 700 | 0 | 0.516304 | 0.000662 | 0.000683 |
| 0.00001 | 700 | 0.1 | 0.516304 | 0.000662 | 0.000683 |
| 0.00002 | 400 | 0.3 | 0.527174 | 0.00064 | 0.000674 |
| 0.00002 | 400 | 0 | 0.538043 | 0.000625 | 0.000671 |
| 0.00002 | 400 | 0.1 | 0.538043 | 0.000625 | 0.000671 |
| 0.00002 | 400 | 0.2 | 0.527174 | 0.000619 | 0.000652 |
| 0.00001 | 400 | 0.3 | 0.527174 | 0.000611 | 0.000644 |
| 0.00001 | 400 | 0 | 0.538043 | 0.000586 | 0.00063 |
| 0.00001 | 400 | 0.1 | 0.538043 | 0.000586 | 0.00063 |
| 0.00001 | 400 | 0.2 | 0.527174 | 0.000585 | 0.000616 |
| 0.00007 | 400 | 0.9 | 0.065217 | 0.005017 | 0.000608 |
| 0.00007 | 700 | 0.9 | 0.065217 | 0.005017 | 0.000608 |
| 0.00007 | 900 | 0.9 | 0.065217 | 0.005017 | 0.000608 |
