# Supplementary material for: An integrated method for the identification of novel genes related to oral cancer
Source: PLoS One. 2017 Apr 6;12(4):e0175185. doi: 10.1371/journal.pone.0175185 (PMC5383255; doi:10.1371/journal.pone.0175185)
Supplement: S3 Table — (DOCX) [file pone.0175185.s004.docx]

**S3 Table.** The performance of the SP-based method with different combinations of parameters

| **Threshold parameter of maximum interaction score $p_{SP}^{MIS}$** | **Threshold parameter of maximum function score $p_{SP}^{MFS}$** | **Recall** | **Precision** | **F1-measure-R** |
| --- | --- | --- | --- | --- |
| 400 | 0.8 | 0.048913 | 0.002917 | 0.000269315 |
| 700 | 0.8 | 0.048913 | 0.002917 | 0.000269315 |
| 900 | 0.8 | 0.048913 | 0.002917 | 0.000269315 |
| 900 | 0.7 | 0.065217 | 0.002054 | 0.000259759 |
| 400 | 0.7 | 0.065217 | 0.002049 | 0.000259107 |
| 700 | 0.7 | 0.065217 | 0.002049 | 0.000259107 |
| 900 | 0.6 | 0.065217 | 0.001262 | 0.000161543 |
| 400 | 0.6 | 0.065217 | 0.001237 | 0.000158282 |
| 700 | 0.6 | 0.065217 | 0.001237 | 0.000158282 |
| 900 | 0.5 | 0.065217 | 0.001075 | 0.000137934 |
| 700 | 0.5 | 0.065217 | 0.001039 | 0.000133434 |
| 400 | 0.9 | 0.016304 | 0.005435 | 0.00013291 |
| 700 | 0.9 | 0.016304 | 0.005435 | 0.00013291 |
| 900 | 0.9 | 0.016304 | 0.005435 | 0.00013291 |
| 400 | 0.5 | 0.065217 | 0.001023 | 0.000131412 |
| 900 | 0.4 | 0.065217 | 0.000887 | 0.000114195 |
| 700 | 0.4 | 0.065217 | 0.00084 | 0.00010813 |
| 400 | 0.4 | 0.065217 | 0.000829 | 0.00010676 |
| 900 | 0.3 | 0.065217 | 0.000791 | 0.000101869 |
| 900 | 0.2 | 0.065217 | 0.000745 | 9.61299E-05 |
| 700 | 0.3 | 0.065217 | 0.000736 | 9.48907E-05 |
| 900 | 0 | 0.065217 | 0.000721 | 9.29994E-05 |
| 900 | 0.1 | 0.065217 | 0.000721 | 9.29994E-05 |
| 400 | 0.3 | 0.065217 | 0.000711 | 9.18255E-05 |
| 700 | 0.2 | 0.065217 | 0.000689 | 8.88908E-05 |
| 400 | 0.2 | 0.065217 | 0.000654 | 8.45212E-05 |
| 700 | 0 | 0.065217 | 0.000654 | 8.45212E-05 |
| 700 | 0.1 | 0.065217 | 0.000654 | 8.45212E-05 |
| 400 | 0 | 0.065217 | 0.000617 | 7.97604E-05 |
| 400 | 0.1 | 0.065217 | 0.000617 | 7.97604E-05 |
