# Supplementary material for: An integrated method for the identification of novel genes related to oral cancer
Source: PLoS One. 2017 Apr 6;12(4):e0175185. doi: 10.1371/journal.pone.0175185 (PMC5383255; doi:10.1371/journal.pone.0175185)
Supplement: S4 Table — (DOCX) [file pone.0175185.s005.docx]

**S4 Table.** Genes identified by the optimal RWR-based method.

1. Genes identified by the optimal RWR-based method with $p_{\text{RWR}}^{MIS}=400$

| **Ensembl ID** | **Probability** | **P-value** | ***MIS*_RWR_** | ***MFS*_RWR_** |
| --- | --- | --- | --- | --- |
| ENSP00000354394 | 0.000436 | <0.001 | 999 | 0.852419 |
| ENSP00000269305 | 0.000349 | 0.015 | 999 | 0.831682 |
| ENSP00000222382 | 0.000335 | <0.001 | 958 | 0.988107 |
| ENSP00000337915 | 0.000332 | <0.001 | 963 | 0.940523 |
| ENSP00000206249 | 0.000282 | <0.001 | 999 | 0.806026 |
| ENSP00000309845 | 0.000279 | 0.023 | 999 | 0.807618 |
| ENSP00000350941 | 0.000256 | 0.014 | 999 | 0.844429 |
| ENSP00000261769 | 0.000234 | <0.001 | 978 | 0.831084 |
| ENSP00000265171 | 0.000224 | <0.001 | 999 | 0.874291 |
| ENSP00000360266 | 0.000221 | <0.001 | 998 | 0.826453 |
| ENSP00000367207 | 0.000214 | 0.039 | 999 | 0.840594 |
| ENSP00000221930 | 0.000212 | 0.001 | 997 | 0.83175 |
| ENSP00000011653 | 0.000206 | 0.009 | 995 | 0.826259 |
| ENSP00000229239 | 0.000202 | <0.001 | 945 | 0.823902 |
| ENSP00000263341 | 0.000192 | <0.001 | 994 | 0.87266 |
| ENSP00000356438 | 0.000191 | 0.008 | 984 | 0.863622 |
| ENSP00000344456 | 0.000186 | 0.001 | 999 | 0.827798 |
| ENSP00000215832 | 0.000185 | 0.006 | 997 | 0.806653 |
| ENSP00000244741 | 0.000175 | 0.002 | 999 | 0.880893 |
| ENSP00000229135 | 0.00017 | 0.03 | 994 | 0.839288 |
| ENSP00000296871 | 0.000168 | 0.005 | 991 | 0.875129 |
| ENSP00000346839 | 0.000167 | 0.015 | 996 | 0.840098 |
| ENSP00000311032 | 0.000159 | 0.006 | 999 | 0.806956 |
| ENSP00000302665 | 0.00015 | <0.001 | 984 | 0.821272 |
| ENSP00000371067 | 0.000136 | 0.007 | 999 | 0.850308 |
| ENSP00000361850 | 0.000133 | <0.001 | 974 | 0.81929 |
| ENSP00000384273 | 0.000132 | 0.041 | 999 | 0.807355 |
| ENSP00000231449 | 0.00013 | 0.011 | 993 | 0.852372 |
| ENSP00000416330 | 0.00013 | 0.001 | 954 | 0.850311 |
| ENSP00000264498 | 0.000124 | 0.016 | 999 | 0.869878 |
| ENSP00000222390 | 0.000124 | 0.013 | 984 | 0.828364 |
| ENSP00000289153 | 0.000123 | <0.001 | 997 | 0.926358 |
| ENSP00000352121 | 0.000121 | <0.001 | 996 | 0.920743 |
| ENSP00000225831 | 0.000121 | 0.012 | 984 | 0.869 |
| ENSP00000366563 | 0.000115 | <0.001 | 997 | 0.918515 |
| ENSP00000263339 | 0.000111 | <0.001 | 976 | 0.814179 |
| ENSP00000304283 | 0.000108 | 0.006 | 990 | 0.980622 |
| ENSP00000363548 | 0.000108 | 0.02 | 999 | 0.849827 |
| ENSP00000313419 | 0.000108 | 0.011 | 925 | 0.836833 |
| ENSP00000228280 | 0.000105 | 0.026 | 948 | 0.815719 |
| ENSP00000359663 | 0.000103 | <0.001 | 999 | 0.838999 |
| ENSP00000292303 | 0.000101 | 0.003 | 996 | 0.839013 |
| ENSP00000223095 | 9.90E-05 | 0.002 | 991 | 0.820027 |
| ENSP00000246657 | 9.82E-05 | <0.001 | 998 | 0.822758 |
| ENSP00000369293 | 9.75E-05 | 0.002 | 999 | 0.866175 |
| ENSP00000324648 | 9.73E-05 | <0.001 | 962 | 0.942562 |
| ENSP00000331736 | 9.73E-05 | <0.001 | 978 | 0.85214 |
| ENSP00000162749 | 9.69E-05 | 0.013 | 999 | 0.824824 |
| ENSP00000260010 | 9.68E-05 | 0.002 | 968 | 0.888427 |
| ENSP00000245451 | 9.57E-05 | 0.026 | 981 | 0.90541 |
| ENSP00000228872 | 9.54E-05 | <0.001 | 999 | 0.818929 |
| ENSP00000360372 | 9.32E-05 | <0.001 | 962 | 0.96635 |
| ENSP00000225474 | 9.27E-05 | <0.001 | 917 | 0.829353 |
| ENSP00000410294 | 9.04E-05 | 0.021 | 999 | 0.845876 |
| ENSP00000365380 | 8.91E-05 | 0.015 | 996 | 0.805597 |
| ENSP00000301141 | 8.85E-05 | <0.001 | 950 | 0.948162 |
| ENSP00000302564 | 8.76E-05 | 0.014 | 999 | 0.823259 |
| ENSP00000245479 | 8.38E-05 | 0.007 | 941 | 0.812842 |
| ENSP00000168712 | 8.21E-05 | 0.001 | 999 | 0.847321 |
| ENSP00000260227 | 7.74E-05 | <0.001 | 972 | 0.805185 |
| ENSP00000356346 | 7.73E-05 | 0.049 | 983 | 0.826366 |
| ENSP00000293272 | 7.70E-05 | 0.002 | 994 | 0.890973 |
| ENSP00000216797 | 7.56E-05 | 0.026 | 999 | 0.824923 |
| ENSP00000228534 | 7.56E-05 | 0.001 | 992 | 0.803231 |
| ENSP00000360247 | 7.48E-05 | 0.004 | 912 | 0.968747 |
| ENSP00000360968 | 7.43E-05 | 0.018 | 939 | 0.958883 |
| ENSP00000308208 | 7.23E-05 | <0.001 | 999 | 0.837376 |
| ENSP00000262158 | 7.21E-05 | <0.001 | 995 | 0.833868 |
| ENSP00000379110 | 6.98E-05 | 0.01 | 955 | 0.826754 |
| ENSP00000355069 | 6.95E-05 | 0.023 | 938 | 0.830027 |
| ENSP00000260356 | 6.94E-05 | <0.001 | 984 | 0.806543 |
| ENSP00000351671 | 6.69E-05 | 0.003 | 965 | 0.804089 |
| ENSP00000379625 | 6.67E-05 | 0.032 | 999 | 0.879566 |
| ENSP00000363081 | 6.66E-05 | 0.021 | 960 | 0.84098 |
| ENSP00000286758 | 6.63E-05 | 0.006 | 992 | 0.837315 |
| ENSP00000259206 | 6.51E-05 | 0.008 | 937 | 0.836138 |
| ENSP00000266646 | 6.44E-05 | 0.028 | 967 | 0.847046 |
| ENSP00000216341 | 6.38E-05 | 0.025 | 991 | 0.808015 |
| ENSP00000332353 | 6.28E-05 | 0.043 | 998 | 0.83623 |
| ENSP00000284523 | 6.19E-05 | 0.043 | 977 | 0.858509 |
| ENSP00000234091 | 6.19E-05 | 0.019 | 960 | 0.807471 |

1. Genes identified by the optimal RWR-based method with $p_{\text{RWR}}^{MIS}=700$

| **Ensembl ID** | **Probability** | **P-value** | **Maximum interaction score** | **Maximum function score** |
| --- | --- | --- | --- | --- |
| ENSP00000354394 | 0.000436 | <0.001 | 999 | 0.852419 |
| ENSP00000269305 | 0.000349 | 0.015 | 999 | 0.831682 |
| ENSP00000222382 | 0.000335 | <0.001 | 958 | 0.988107 |
| ENSP00000337915 | 0.000332 | <0.001 | 963 | 0.940523 |
| ENSP00000206249 | 0.000282 | <0.001 | 999 | 0.806026 |
| ENSP00000309845 | 0.000279 | 0.023 | 999 | 0.807618 |
| ENSP00000350941 | 0.000256 | 0.014 | 999 | 0.844429 |
| ENSP00000261769 | 0.000234 | <0.001 | 978 | 0.831084 |
| ENSP00000265171 | 0.000224 | <0.001 | 999 | 0.874291 |
| ENSP00000360266 | 0.000221 | <0.001 | 998 | 0.826453 |
| ENSP00000367207 | 0.000214 | 0.039 | 999 | 0.840594 |
| ENSP00000221930 | 0.000212 | 0.001 | 997 | 0.83175 |
| ENSP00000011653 | 0.000206 | 0.009 | 995 | 0.826259 |
| ENSP00000229239 | 0.000202 | <0.001 | 945 | 0.823902 |
| ENSP00000263341 | 0.000192 | <0.001 | 994 | 0.87266 |
| ENSP00000356438 | 0.000191 | 0.008 | 984 | 0.863622 |
| ENSP00000344456 | 0.000186 | 0.001 | 999 | 0.827798 |
| ENSP00000215832 | 0.000185 | 0.006 | 997 | 0.806653 |
| ENSP00000244741 | 0.000175 | 0.002 | 999 | 0.880893 |
| ENSP00000229135 | 0.00017 | 0.03 | 994 | 0.839288 |
| ENSP00000296871 | 0.000168 | 0.005 | 991 | 0.875129 |
| ENSP00000346839 | 0.000167 | 0.015 | 996 | 0.840098 |
| ENSP00000311032 | 0.000159 | 0.006 | 999 | 0.806956 |
| ENSP00000302665 | 0.00015 | <0.001 | 984 | 0.821272 |
| ENSP00000371067 | 0.000136 | 0.007 | 999 | 0.850308 |
| ENSP00000361850 | 0.000133 | <0.001 | 974 | 0.81929 |
| ENSP00000384273 | 0.000132 | 0.041 | 999 | 0.807355 |
| ENSP00000416330 | 0.00013 | 0.001 | 954 | 0.850311 |
| ENSP00000231449 | 0.00013 | 0.011 | 993 | 0.852372 |
| ENSP00000222390 | 0.000124 | 0.013 | 984 | 0.828364 |
| ENSP00000264498 | 0.000124 | 0.016 | 999 | 0.869878 |
| ENSP00000289153 | 0.000123 | <0.001 | 997 | 0.926358 |
| ENSP00000352121 | 0.000121 | <0.001 | 996 | 0.920743 |
| ENSP00000225831 | 0.000121 | 0.012 | 984 | 0.869 |
| ENSP00000366563 | 0.000115 | <0.001 | 997 | 0.918515 |
| ENSP00000263339 | 0.000111 | <0.001 | 976 | 0.814179 |
| ENSP00000363548 | 0.000108 | 0.02 | 999 | 0.849827 |
| ENSP00000313419 | 0.000108 | 0.011 | 925 | 0.836833 |
| ENSP00000304283 | 0.000108 | 0.006 | 990 | 0.980622 |
| ENSP00000228280 | 0.000105 | 0.026 | 948 | 0.815719 |
| ENSP00000359663 | 0.000103 | <0.001 | 999 | 0.838999 |
| ENSP00000292303 | 0.000101 | 0.003 | 996 | 0.839013 |
| ENSP00000223095 | 9.90E-05 | 0.002 | 991 | 0.820027 |
| ENSP00000246657 | 9.82E-05 | <0.001 | 998 | 0.822758 |
| ENSP00000369293 | 9.75E-05 | 0.002 | 999 | 0.866175 |
| ENSP00000331736 | 9.73E-05 | <0.001 | 978 | 0.85214 |
| ENSP00000324648 | 9.73E-05 | <0.001 | 962 | 0.942562 |
| ENSP00000162749 | 9.69E-05 | 0.013 | 999 | 0.824824 |
| ENSP00000260010 | 9.68E-05 | 0.002 | 968 | 0.888427 |
| ENSP00000245451 | 9.57E-05 | 0.026 | 981 | 0.90541 |
| ENSP00000228872 | 9.54E-05 | <0.001 | 999 | 0.818929 |
| ENSP00000360372 | 9.32E-05 | <0.001 | 962 | 0.96635 |
| ENSP00000225474 | 9.27E-05 | <0.001 | 917 | 0.829353 |
| ENSP00000410294 | 9.04E-05 | 0.021 | 999 | 0.845876 |
| ENSP00000365380 | 8.91E-05 | 0.015 | 996 | 0.805597 |
| ENSP00000301141 | 8.85E-05 | <0.001 | 950 | 0.948162 |
| ENSP00000302564 | 8.76E-05 | 0.014 | 999 | 0.823259 |
| ENSP00000245479 | 8.38E-05 | 0.007 | 941 | 0.812842 |
| ENSP00000168712 | 8.21E-05 | 0.001 | 999 | 0.847321 |
| ENSP00000260227 | 7.74E-05 | <0.001 | 972 | 0.805185 |
| ENSP00000356346 | 7.73E-05 | 0.049 | 983 | 0.826366 |
| ENSP00000293272 | 7.70E-05 | 0.002 | 994 | 0.890973 |
| ENSP00000228534 | 7.56E-05 | 0.001 | 992 | 0.803231 |
| ENSP00000216797 | 7.56E-05 | 0.026 | 999 | 0.824923 |
| ENSP00000360247 | 7.48E-05 | 0.004 | 912 | 0.968747 |
| ENSP00000360968 | 7.43E-05 | 0.018 | 939 | 0.958883 |
| ENSP00000308208 | 7.23E-05 | <0.001 | 999 | 0.837376 |
| ENSP00000262158 | 7.21E-05 | <0.001 | 995 | 0.833868 |
| ENSP00000379110 | 6.98E-05 | 0.01 | 955 | 0.826754 |
| ENSP00000355069 | 6.95E-05 | 0.023 | 938 | 0.830027 |
| ENSP00000260356 | 6.94E-05 | <0.001 | 984 | 0.806543 |
| ENSP00000351671 | 6.69E-05 | 0.003 | 965 | 0.804089 |
| ENSP00000379625 | 6.67E-05 | 0.032 | 999 | 0.879566 |
| ENSP00000363081 | 6.66E-05 | 0.021 | 960 | 0.84098 |
| ENSP00000286758 | 6.63E-05 | 0.006 | 992 | 0.837315 |
| ENSP00000259206 | 6.51E-05 | 0.008 | 937 | 0.836138 |
| ENSP00000266646 | 6.44E-05 | 0.028 | 967 | 0.847046 |
| ENSP00000216341 | 6.38E-05 | 0.025 | 991 | 0.808015 |
| ENSP00000332353 | 6.28E-05 | 0.043 | 998 | 0.83623 |
| ENSP00000284523 | 6.19E-05 | 0.043 | 977 | 0.858509 |
| ENSP00000234091 | 6.19E-05 | 0.019 | 960 | 0.807471 |
